# Cannabis and Its Secondary Metabolites: Their Use as Therapeutic Drugs, Toxicological Aspects, and Analytical Determination

**DOI:** 10.3390/medicines6010031

**Published:** 2019-02-23

**Authors:** Joana Gonçalves, Tiago Rosado, Sofia Soares, Ana Y. Simão, Débora Caramelo, Ângelo Luís, Nicolás Fernández, Mário Barroso, Eugenia Gallardo, Ana Paula Duarte

**Affiliations:** 1 Centro de Investigação em Ciências da Saúde, Faculdade de Ciências da Saúde da Universidade da Beira Interior (CICS-UBI), 6200-506 Covilhã, Portugal; janitagoncalves@hotmail.com (J.G.); tiagorosadofful@hotmail.com (T.R.); sofia_soares_26@hotmail.com (S.S.); anaaysa95@gmail.com (A.Y.S.); deboracaramela50@gmail.com (D.C.); afluis27@gmail.com (Â.L.); egallardo@fcsaude.ubi.pt (E.G.); 2 Universidad de Buenos Aires, Facultad de Farmacia y Bioquímica, Cátedra de Toxicología y Química Legal, Laboratorio de Asesoramiento Toxicológico Analítico (CENATOXA). Junín 956 7mo piso. Ciudad Autónoma de Buenos Aires (CABA), Buenos Aires C1113AAD, Argentina; nfernandez@ffyb.uba.ar; 3 Serviço de Química e Toxicologia Forenses, Instituto de Medicina Legal e Ciências Forenses - Delegação do Sul, 1169-201 Lisboa, Portugal; mario.j.barroso@inmlcf.mj.pt

**Keywords:** cannabis, cannabinoids, therapeutics, toxicology, analytical determination, legalization

## Abstract

Although the medicinal properties of *Cannabis* species have been known for centuries, the interest on its main active secondary metabolites as therapeutic alternatives for several pathologies has grown in recent years. This potential use has been a revolution worldwide concerning public health, production, use and sale of cannabis, and has led inclusively to legislation changes in some countries. The scientific advances and concerns of the scientific community have allowed a better understanding of cannabis derivatives as pharmacological options in several conditions, such as appetite stimulation, pain treatment, skin pathologies, anticonvulsant therapy, neurodegenerative diseases, and infectious diseases. However, there is some controversy regarding the legal and ethical implications of their use and routes of administration, also concerning the adverse health consequences and deaths attributed to marijuana consumption, and these represent some of the complexities associated with the use of these compounds as therapeutic drugs. This review comprehends the main secondary metabolites of *Cannabis*, approaching their therapeutic potential and applications, as well as their potential risks, in order to differentiate the consumption as recreational drugs. There will be also a focus on the analytical methodologies for their analysis, in order to aid health professionals and toxicologists in cases where these compounds are present.

## 1. Introduction

The secondary metabolism of plants plays an important role in their survival in its environment. Secondary metabolites are able to attract pollinators, to defend plants against predators and diseases, and for that reason have been exploited for biopharmaceutical purposes. The secondary metabolites are also present in great amounts in the so-called food plants, conferring them taste, color, and scent. Moreover, numerous plant secondary metabolites such as alkaloids, anthocyanins, flavonoids, quinones, lignans, steroids, and terpenoids have found commercial applications, namely as drugs, dye, flavor, fragrance, and insecticides [1]. 

*Cannabis sativa* L. (*Cannabinaceae*), also known as marijuana or hemp, belongs to a group of herbaceous shrubs 1 to 2 m in height, and is widely distributed in temperate and tropical areas. Three species are usually recognized: *Cannabis sativa*, *Cannabis indica*, and *Cannabis ruderalis* (the latter may be included under *C. sativa*), and all may be treated as subspecies of a single species, *C. sativa*. The plant is accepted as being native from Central Asia. Several preparations of *C. sativa* including marijuana, hashish, charas, dagga, and bhang, are estimated to be consumed by 200–300 million people around the world [2], being the most popular illicit drug of the 21st century according to the United Nation Office on Drugs and Crime (UNODC) [3].

*Cannabis* has been cultivated widely in the world for its achene fruits (often wrongly referred to as seeds), which are rich in oils and other phytonutrients, and are frequently used as human food or animal feedstuff, as well as for its fibers, for traditional medicine and spiritual purposes as therapeutic and hallucinogenic drug [2,4]. *Cannabis* is one of the most consumed drugs worldwide, together with legal drugs such as tobacco, alcohol, and caffeine.

*Cannabis* plants contain more than 545 known compounds. In addition to phytocannabinoids (which are C_21_ terpenophenolic, or C_22_ for the carboxylated forms, compounds with physiological and often psychotogenic effects, possessing monoterpene and alkylresorcinol moieties in their molecules [4,5,6]), they include alkanes, sugars, nitrogenous compounds (such as spermidine alkaloids or muscarine), flavonoids, non-cannabinoid phenols, phenylpropanoids, steroids, fatty acids, approximately 140 different terpenes that are predominantly monoterpenes such as β-myrcene, α- and β-pinene, α-terpinolene, but also sesquiterpenes including β-caryophyllene, di- and triterpenes, as well as various other common compounds [4,5].

**Figure 1:**
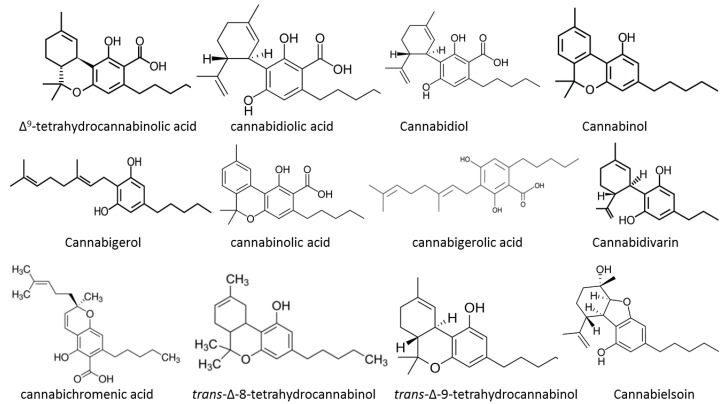
Some natural cannabinoids from the cannabis plant.

Out of over 100 cannabinoids identified so far, the most potent in terms of psychoactive activity is *trans*-Δ-9-tetrahydrocannabinol (THC) [7]. Four stereoisomers of THC exist, but only the (–)-trans isomer occurs naturally. Two structurally related substances (Δ9-tetrahydrocannabinol-2-oic acid and Δ9-tetrahydrocannabinol-4-oic acid - THCA) are usually also present, sometimes in large amounts. The heat of combustion during smoking converts partly THCA to THC. One isomer which is also active (Δ8-THC) occurs in much smaller amounts. Other related compounds include cannabidiol (CBD) and cannabinol (CBN), the latter particularly in aged samples, and presenting pharmacological effects different than those attributed to THC (Figure 1). All these compounds are collectively known as cannabinoids and, unlike many other psychoactive substances, are not nitrogenous bases [8]. Individual cannabinoids are being developed and identified in *Cannabis* strains, and their effects on symptoms of illnesses suffered by patients are being studied. The main types of natural cannabinoids belong to the families of the cannabigerol-type, cannabichromene-type, cannabidiol-type, cannabinodiol-type, tetrahydrocannabinol-type, cannabinol-type, cannabitriol-type, cannabielsoin-type, isocannabinoids, cannabicyclol-type, cannabicitran-type, and cannabichromanone-type [9].

THC has been used as an anti-vomiting drug in cancer chemotherapy and as an appetite stimulant, especially for AIDS patients [2]. On the other hand, CBD, the isomer of THC, has no psychotropic effect. However, it possesses a variety of other pharmacological activities [2], namely, it reduces aggressive behavior in the L-pyroglutamate-treated rat, spontaneous dyskinesias in the dystonic rat, and turning behavior in the 6-hydroxyldopamine-treated rat caused by apomorphine [2]. Cannabichromene and related compounds possess anti-inflammatory, anti-fungal, and anti-microbial activities [2]. Therefore, cannabinoids are considered to be promising agents for the treatment of several types of diseases [2].

Based on the content of the psychoactive constituent THC, the chemotypes of *C. sativa* include the drug type (marijuana, 1.0–20% THC), intermediate type (0.3–1.0% THC), and fiber type (hemp, <0.3% THC) [10,11]. The drug type is regarded as an illicit drug of abuse and its cultivation is prohibited in most nations due to the psychotropic effect [10]. While the fiber type (hemp), cultivated as a source of textiles and food, is legal in several countries [10]. 

Cannabinoids are synthesized and stored predominantly in glandular trichomes, hair-like epidermal protrusions densely concentrated in the bracts and flowers of cannabis plants [12]. Various strategies have been pursued to extract and deliver the pharmacological agents from cannabis. The use of chemical solvents such as petroleum ether or ethanol are likely to leave unwanted residues, whereas extractants such as olive or coconut oil provide a more organic alternative [12].

The therapeutic potential and applications of those compounds, as well as their potential risks, in order to differentiate from recreational consumption will be discussed below. In addition, a focus on the analytical methodologies for their analysis will be presented.

## 2. Modes of Use in Recreational and Therapeutic Situations, Pharmacokinetics, and Pharmacodynamics

Despite the increasing interest that cannabinoids have aroused, their pharmacokinetics and pharmacodynamics are not yet fully understood, limiting the work of researchers [13]. Regarding the cannabinoids with therapeutic applications, the information about these concepts is even less abundant [13]. The pharmacokinetics of these compounds are strongly related to their route of administration [14,15,16]. The route of consumption of cannabinoids most commonly used is the airways. Smoking is more common for recreational consumption, while the vaporized form is used for both recreational and therapeutic purposes [13]. However, the trends of consumption of cannabis as recreational use change quickly, namely with the advent of new synthetic substances, as synthetic cannabinoids [17]. 

The use of the respiratory tract as a form of consumption allows a rapid and efficient passage from the lungs to the brain [18]. Plasma concentrations of THC and CBD are detected within a few seconds of inhalation, reaching their maximum 3 to 10 min after consumption [19,20,21,22]. In a study by Kauert et al. [23], it was found that the maximum concentrations of serum THC after smoking cigarettes with about 18.2 mg and 36.5 mg of cannabinoids were 48 μg/L and 79 μg/L, respectively. The bioavailability of CBD is about 31% and ranges from 10% to 35% for THC [20,21]. The large range of variation is due not only to the variability within subjects, but also to inhalation time, number, interval, and duration of puffs, inhalation volume, and particle size [15,24]. The inhaled device also interferes with these values because when using tobacco, part of the active compound present in the cigarette is destroyed by pyrolysis (about 23% to 30%) [25]. There are also losses at the end of the cigarette and second-hand smoke since they are not consumed [25]. It should be emphasized that the bioavailability values vary between smokers and non-smokers. Studies by Lindgren et al. [26] and by Azorloza et al. [27] allowed to verify that the bioavailability of THC in smokers (23% to 27%) is superior to the bioavailability in non-smokers (10% to 14%).

Another common way to consume cannabinoids, both therapeutic and recreational, is oral administration in the form of capsules, food or cannabis-infused drink [18]. This route has the advantage of not forming harmful compounds during consumption, as it happens when smoked [18]. By oral consumption, both THC and CBD have reduced bioavailability (less than 20%), since they are highly lipophilic [28,29,30]. In a study by Ohlsson et al. [31] a bioavailability of 6 ± 3% was obtained for the ingestion of 20 mg of THC in a chocolate biscuit. However, in another study by Wall et al. [32], after ingestion of cannabinoids in gelatine capsules, the bioavailability obtained was 10% to 20%. Absorption is variable, as the compounds are degraded either in the stomach or in the intestines [33]. In areas where pH is low, isomerizations and protonations may occur, resulting in substituted CBDs [33]. This route of administration causes extensive hepatic metabolism to the ingested compounds, with maximum plasma concentrations of THC and CBD being reached between 1 h and 2 h after the consumption [13,20]. Nevertheless, there are studies where these same concentrations were only reached at 4 h [34] and 6 h [26,35] after consumption. In 2015, Ahmed et al. [36] administered orally 0.75 mg and 1.5 mg in elderly patients, with a maximum concentration of 0.41 μg/L and 1 μg/L, respectively. However, in another study, where 20 mg of cannabinoids were administered orally in daily users, the maximum concentrations obtained were 16.5 μg/L [37]. 

One variation of oral administration is the use of oromucosal delivery. This route of administration is mainly used for therapeutic applications [13]. Absorption occurs rapidly through the buccal mucosa, reaching higher plasma concentrations when compared to the oral route of administration [13]. Sublingual administration is also common for therapeutic use [20]. By using this route of administration, first-pass hepatic metabolism is avoided [18]. In a phase II study where THC was administered, it was possible to measure plasma concentrations of 14 μg/L [38].

The dermal administration of cannabinoids is a pathway that has been used for therapeutic purposes [20]. The application of compounds in the skin also avoids the first pass metabolism [18]. Cannabinoids have a hydrophobic nature, limiting its diffusion through the skin [13]. Studies on human skin have shown that CBD crosses the skin barrier more easily than THC, since it is more lipophilic [39,40]. Another study in guinea pigs showed that the plasma concentration of THC was 4.4 ng/mL at 1.4 h and was maintained for more than 48 h [41]. There are also studies in which the ways of improving the bioavailability of cannabinoids using this route of administration have been explored [20,40,42].

Other less common routes that have been investigated for therapeutic purposes are rectal and ophthalmic [20]. During rectal administration, the bioavailability of cannabinoids is very discrepant, depending on the composition of the suppository [20]. A THC hemisuccinate suppository was the one with the highest bioavailability (13.5%) in monkeys [43]. In a study by Brenneisen et al. [44], where THC plasma concentrations were evaluated, it was found that after administration of 2.5 to 5 mg of THC, peak plasma concentrations ranged from 1.1 to 4.1 ng/mL and were reached between 2 h and 8 h, respectively. Regarding the ophthalmic route, there is a much smaller number of studies. A study in rabbits showed very different bioavailability values (6% to 40%). It was also possible to verify that maximum plasma concentrations were reached after 1 h of administration [45].

After absorption, the cannabinoids decrease their concentration in the plasma, as they are distributed rapidly by the tissues [18]. This distribution is based on the physical-chemical properties of cannabinoids and the degree of the tissue irrigation [13,20]. Also, individual characteristics, such as body composition and health status, can influence this process [46]. Thus, cannabinoids distribute more rapidly through more irrigated tissues, such as the brain, lung, heart and liver [13,47,48]. Distribution volumes are estimated approximately 3.4 L/kg for THC and 32 L/kg for CBD [21,49]. Due to their lipophilicity, these compounds have high affinity for adipose tissue, and their chronic consumption can lead to accumulation in that tissue with subsequent redistribution [13,20].

In the particular case of cannabinoid consumption during pregnancy, THC crosses the placenta rapidly and can reach the foetus [30,50]. Studies have revealed that maternal blood THC concentrations were very close to the concentrations found in foetal blood, although foetal blood concentrations were lower [20]. Chronic cannabinoid consumption also leads to accumulation of THC in breast milk [20]. A human study showed that the concentration of THC in breast milk was higher than the concentration of the same compound in plasma [51]. In this way, it was verified that a child in the stage of breast-feeding consumes daily between 0.01 and 0.1 mg of THC, for each one or two cigarettes of cannabis that the mother smokes [20].

**Figure 2:**
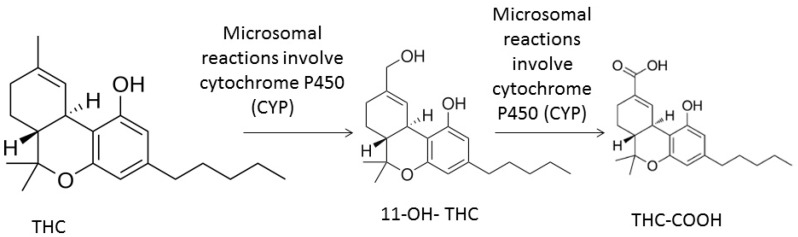
Main metabolites of THC.

The metabolism of THC is mainly at the hepatic level and involves phase I reactions: aliphatic hydroxylations, oxidation of alcohols to ketones and acids, β-oxidation and degradation of the pentyl lateral chain, epoxides, decarboxylations, and conjugations [18,20,52]. Microsomal reactions involve cytochrome P450 (CYP) complex enzymes [53,54]. In humans the CYP2C9, CYP2C19, and CYP3A4 subfamilies are responsible for the metabolism of THC in the liver [13,20,55]. However, extrahepatic metabolism also occurs in tissues expressing CYP450 complex enzymes, such as the brain, small intestine, heart, and lung [13,20]. More than 100 THC metabolites were identified, which are mostly mono-hydroxylated compounds [20,25]. In humans, C-11 is the most attacked site, being the major metabolites 11-hydroxy-THC (11–OH–THC, active) and 11-carboxy-THC (THC–COOH, inactive) (Figure 2) [20,21,52,56]. Huestis et al. [57] detected peak concentrations of 11–OH–THC only 13 min after the onset of smoking. Subsequently, these metabolites undergo glucuronidation as a phase II reaction, or, less commonly, conjugation with amino acids, fatty acids, sulphate and glutathione [13,18,20]. These reactions are catalysed by UGT1A9 and UGT1A10 in the case of 11–OH–THC and UGT1A1 and UGT1A3 in the case of THC-COOH [30]. Also, C-8 and C-9 are attacked, but in a smaller scale [18,20].

The metabolism of CBD and CBN is similar to that of THC [18,20]. Concerning CBD, it also occurs at the hepatic level and is firstly performed by the CYP2C19 and CYP3A4 subfamilies and subsequently by the CYP1A1, CYP1A2, CYP2C9, and CYP2D6 subfamilies [13,58]. Oxidation reactions occur at C-9 and the lateral chain, but a part of this compound is excreted unchanged [18]. As regards CBN, a primary metabolite is formed by hydroxylation of C-9 to form an additional aromatic ring [18]. Thus, this compound is metabolized more slowly and less extensively than THC [59].

After metabolism, the plasma concentrations of cannabinoids and their metabolites gradually decrease. In a study by Huestis et al. [22], the concentration of 0.5 μg/L of THC in the plasma took between 3 h and 12 h to be reached after smoking a cigarette with 16 mg of THC. When consuming a dose of 34 mg, the same concentration took between 6 and 27 h to be reached. In the same study, THC-COOH was detected between 2 and 7 days at the lowest dose and between 3 and 7 days after administration of the highest dose [22]. The elimination half-life of THC is not easy to calculate because it is influenced by the balance between plasma and adipose tissue, which is a time-consuming process [13,20]. Also, CBD presents a great variation as regards its half-life time. In a study by Consroe et al. [60], it was possible to verify that after daily oral intake of CBD, elimination half-life ranged from 2 to 50 days. In another work by Ohlsson et al. [21] a mean elimination half-life of 31 ± 4 h after inhalation of CBD was reported.

Cannabinoids after being metabolized are excreted for days [61]. Typically, between 80% and 90% of the THC consumed is excreted as carboxylate and hydroxylate metabolites [18,62,63]. Approximately 5% of the acid metabolites are eliminated unchanged, 20% to 35% are excreted in the urine, and between 65% and 80% are eliminated in the faeces [25,32,48]. The major excreted glucuronic metabolite is THC–COOH glucuronide. However, its free form (THC–COOH) is also excreted through the urine [34,64,65]. On the other hand, the predominantly excreted form in faeces is 11–OH–THC [18,63]. The elimination of CBD is similar to the one of THC [20]. About 16% of the metabolites of this compound are excreted via urine in 72 h [20]. However, in the case of CBD a high amount is excreted unchanged in the faeces [20].

Concerning the mechanism of action, the effects of cannabinoids occur through agonism in specific receptors [20]. These receptors are part of the endocannabinoid system, which plays important roles in the development of the central nervous system (CNS), synaptic plasticity, and also in the response to both external and endogenous aggressions. This system is constituted not only by cannabinoid receptors, but by endogenous cannabinoids (endocannabinoids) and the enzymes responsible for their synthesis and degradation as well [66]. Two cannabinoid receptors are identified, CB1 and CB2 [67]. Other entities, as the transient receptor potential (TRP) channels, and peroxisome proliferator activated receptors (PPARs) are also engaged by some cannabinoid’s compounds. The endocannabinoid system also plays an important role in different diseases, for instance in epilepsia. CB1 and CB2 receptors are coupled to inhibitory G proteins, which when activated inhibit adenylate cyclase, inhibiting the conversion of AMP to cyclic-AMP [20]. CB1 receptors are found mainly in the central and peripheral nervous system and also in some peripheral organs and tissues (e.g., leukocytes, spleen, endocrine glands, heart and areas of the reproductive, urinary and gastrointestinal systems) [67]. CB2 receptors are mainly located at the level of the immune system (tonsils, spleen, and leukocytes) and hematopoietic cells, which are of great interest for therapeutic purposes [20,30]. The affinity presented by cannabinoids is different for each type of receptor [20]. THC has affinity for both types of receptors, behaving as a partial agonist of both, yet it is more effective for CB1 type [13,20,68]. It is through this link that THC exerts its psychoactive and analgesic effects [13]. Cannabidiol has low affinity for both types of receptors [69,70]. It has been described that this compound exerts its activity through non-cannabinoid receptors [30,69]. This evidence was verified in a study by Laprairie et al. [69] where CBD was found to modulate CB1R receptors by binding to an allosteric site. TRP channels are located mostly on cell-membranes, and many of them are responsible for mediating several physiological responses, for instance pain, temperature, tastes, pressure, and vision. These channels interact with other proteins, and often form signalling complexes, for which the exact pathways of which are not known [71]. TRP channels, particularly TRPV1, which is located in dorsal root and trigeminal ganglia, brain, peripheral nerve ends, skin, bladder, pancreas and testis, are activated by the endocannabinoid anandamide under certain conditions [72]. On the other hand, PPARs are a group of nuclear receptor proteins that act as transcription factors that regulate gene expression. They play crucial roles in the regulation of cell differentiation, development, metabolism, and tumorigenesis of higher organisms. The different isoforms of PPARs (α, β, and γ) are activated by some cannabinoids, as has been shown in reporter gene assays, binding studies, selective antagonists and knockout studies. Some cannabinoid-induced effects follow the activation of these isoforms, mainly PPARα and γ, including analgesic, neuroprotective, neuronal function modulation, anti-inflammatory, metabolic, anti-tumor, gastrointestinal and cardiovascular effects. This activation often occurs in conjunction with the activation of the more traditional target sites of action, namely the CB1 and CB2 receptors and the TRPV1 ion channel. Some of the effects of inhibitors of endocannabinoid degradation or transport are also mediated by PPARs [73]. 

## 3. Secondary and Toxic Effects, Dependence, and Tolerance

Cannabis smokers usually inhale deeply and hold their breath to maximize THC absorption in the lungs, in order to achieve the desired effects. These effects appear within a few minutes time interval, and one of the most common is euphoria. The sensations of relaxation and pleasure are the main reason for young people to take cannabis, but the intensity of the “high” depends on the dose, mode of administration and the user’s prior experience with cannabis or other drugs (for example, tobacco smokers have more experience compared to people who have never smoked). Besides the known euphoria and therapeutic effects, there are several authors that report effects on memory and cognition, motor function, reaction time, and psychomotor performance. A state of physical inertia with ataxia, dysarthria, and incoordination are some symptoms that can last for a few hours, as well as an increased speed of thought. The time distortion along with poor psychomotor performance for certain tasks can be explained by memory lapses. Physically, the immediate effect that cannabis users present is the increase in blood pressure, which may account for changes in the heart rate. Taking into account the effects usually associated with cannabis consumption, a major concern nowadays is the ability to drive motor vehicles under the influence of such substances. In fact, cannabis use is a major risk for road accidents, even more because it is often used concomitantly with other drugs, such as alcohol; in addition, the effects of both drugs on the psychomotor impairment are additive [74,75].

Regarding acute toxicity, there is no supporting evidence that cannabinoid consumption may induce overdose and/or death situations [76]. However, coma situations have been described in case of ingestion by children [74].

The chronic effects of cannabis are characterized by their complexity, and the literature reports that cannabis users are usually affected in several organic systems (immune, respiratory, gastrointestinal, cardiovascular, and reproductive). Concerning the cardiovascular system, besides the negative effects on the oxygen delivery, smoking cannabis contributes to the increase of carboxyhaemoglobin and heart effort. As mentioned above, elevated blood pressure may lead to strokes in patients with cerebrovascular disease, or to exacerbations in case of hypertensive patients [75]. In contrast, chronic cannabis smokers develop bronchitis and emphysema symptoms, as well as abnormalities in the large airways and poor function of the lungs. Another factor which may contribute to respiratory complications is that cannabis is usually smoked together with tobacco, increasing the risks of lung cancer and other diseases. Regarding the reproductive system, men are affected by a reduction in the number of spermatozoids and decreased sperm mobility, facing the risk of infertility, while in women the effects caused by cannabis-smoking mothers during their pregnancy are not very clear. It is assumable that during the pregnancy, as a result of exposure of the fetus in the uterus, the use of cannabis produces an increased risk of birth defects [74]. Concerning the role of CB2 receptors on the immunosuppressive actions, high doses or concentrations of THC may induce effects on the immunologic system, such as stimulation of the lymphocytes proliferation, enhanced production of interleukin-2, and macrophage function [77]. Cannabis use has also been correlated to the onset of some psychotic episodes. Despite being uncommon, some studies have associated these episodes to heavy cannabis users, in which large doses of THC produced delusions, hallucinations, and paranoid ideations. This so-called “cannabis psychosis” has been reported to cause an exacerbation of the symptoms of mental illness, like schizophrenia. The frequent use and the rising potency of cannabis suggest that young people may have their first-episode of psychosis earlier than usual [74,78]. 

Other disorders related with mood and anxiety have been part of the complications of long-term use of cannabis. Bipolar disorder, panic attacks, and severe anxiety are some effects caused by this chronic cannabis use [79]. 

Tolerance to cannabis is usually associated to effects as antinociception, anticonvulsant activity, loss of locomotor activity, and hypotension caused by repeated doses of THC and other psychoactive cannabinoids. Tolerance to most drugs normally occurs in two ways, either by changes in pharmacokinetics or by variations in pharmacodynamics. In the case of cannabinoids tolerance develops by changes in pharmacodynamics, once there is evidence that the receptors play an important role [77]. Tolerance can affect mood, memory, psychomotor performance, sleep, heart rate, arterial pressure and body temperature, among other symptoms. In several studies the cognitive effects of tolerance were identified, but not much attention was paid to psychopathological effects [74]. These studies play a decisive role in what concerns the therapeutic use of cannabis, which may promote certain problems if drug consumption originates serious manifestations. Furthermore, there is frequent development of tolerance in conjunction with dependence and withdrawal symptoms. 

Dependence and withdrawal symptoms are characterized by a strong desire to reuse the substance and a defective control over its use, and these are indicators that a person is undergoing a drug dependency. Abstinence symptoms occur when there is an interruption in drug use and adverse effects appear due to this abrupt reduction of use [80]. 

For many years, it was common to claim that cannabis did not present dependence, since there was no evidence of tolerance or withdrawal syndrome, particularly when compared to other drugs. 

However, it was observed in some studies that the withdrawal syndrome of cannabis had some similarities to those of alcohol and opioid withdrawal states. Some of the effects related to this dependence include anxiety, irritability, insomnia, muscle tremor, anorexia, and increased reflexes [74,75]. In recent surveys [81], a substantial proportion of people who show dependence are long-term cannabis users, with 57% from 243 users qualified for lifetime DSM-III-R and ICD-10 cannabis dependence diagnoses. On the other hand, Anthony et al. [82] estimated that few persons who had ever used cannabis met the criteria DSM-III-R for dependence on this drug at some time in their lives, which suggests that some chronic cannabis users can develop cannabis dependence syndrome. In fact, most authors claim that long-term cannabis use has been associated with a decline in cognitive functions, which seemingly can be reversed, however, after a few days of abstinence [9,83,84].

Budney et al. [85] reported a comparison of three possible treatments based on motivational strategies. However, it is still not clear what treatment must be provided, if some, for dependent cannabis users that cannot stop using despite of knowing the adverse effects. 

Despite the controversy regarding dependence, relative to other drugs of abuse, cannabis is usually associated with minimal withdrawal symptoms, but with the development of tolerance instead. 

## 4. Prevalence and Control Status

High-risk drug use is one of the five key indicators of the European Monitoring Centre for Drugs and Drug Addiction (EMCDDA) for monitoring in Europe. This type of indicator is an important tool for consumption trends, as well as for drug categorization. As mentioned above, cannabis is the most consumed drug worldwide, which is due in part to the ease of acquisition and to the low prevalence of dependence situations. In the last report of the EMCDDA, in 2017, the drug accounted for 74% of an overall estimate of 1.5 million offences. Cannabis is also the most commonly used illicit drug in Europe, and its prevalence is about five times higher than that of other drugs. It is estimated that 17.2 million young adults (aged 15–34 years) have used cannabis in the last year across the European Union, while 87.6 million will use it throughout life (aged 15–64 years). The trends in use also vary between countries. In surveys performed from around 2017, the prevalence rates in the previous year in the age range of 15 to 34 range from 3.5% in Hungary to 21.5% in France [86,87]. Based on surveys of the general population, it is estimated that around 1% of European adults are daily or almost daily users of cannabis, meaning that they have consumed this drug in 20 or more days from the previous month. About 37% of these people are older consumers, aged 35-64, with about three-quarters of them being male [86,87,88,89].

Concerning regulation issues, cannabis and cannabis resin are both listed in Schedules I and IV of the United Nations 1961 Single Convention on Narcotic Drugs [90]. The debate concerning laws aiming at prohibiting or permitting the use of this drug worldwide has gained new fuel since 2012, as the supply and use of recreational cannabis in Uruguay and some states of the US (and more recently in Canada). Legislative proposals aiming at legalizing cannabis have raised important concerns on the eventual increase in its use, and consequently the related harms, and possibilities related to the ways in which cannabis for non-medical purposes could be regulated to mitigate these concerns have raised. Indeed, the use of cannabis-based products as medicine to treat certain conditions is not prohibited by international law. Also, and according to UN conventions, the use of any drug under international control should be limited to those situations involving medical and scientific purposes. 

The system of controls which is deemed necessary in the case that a country decides to allow cultivation of cannabis not intended for industrial or horticultural purposes is described under Article 28 of the 1961 Convention, while the 1971 Convention controls specifically THC. In Europe, THC may be included in capsules, cannabis extract may be used as a mouth spray, and dried cannabis flowers may be used for vaporizing or making cannabis tea, all of those uses in the case of authorized medicines [86]. By contrast, smoking cannabis for medical purposes is not authorized at all, which is due on the one hand to the fact that there are many strains of cannabis plants, and each one of them has the capability of producing a wide range of chemicals. In addition, the range and concentration of chemicals may also vary within one plant, and it depends for instance on light levels during growth or maturity at harvest. Therefore, these factors should have to be strictly controlled to allow the prescriber and/or the pharmacist to be able to judge the content and as such deliver the needed chemicals for a particular patient. On the other hand, inhaling smoke from burning plant material can hardly be considered a healthy method for the delivery of substances to the bloodstream, as harmful tars and particles will be inhaled as well. Also, when the chemicals have no psychoactive activity, for instance as occurs with CBD, it is difficult for the user to know accurately the dose. There is no harmonized EU law on cannabis use, and each country may deal with drug offences differently [86]. Indeed, while many countries have adopted decriminalization and have turned the simple possession of drugs a non-criminal offense, others have preconize much more severe penalties, and the mere possession of even small amounts of drug can lead to several years in prison [91].

The evolution of the cannabis market, triggered by recent developments on the American continent concerning its legalization in some jurisdictions, entails new policy challenges, as a rapid development of a commercial market for cannabis has emerged. Consequently, innovations concerning the preparations under which the drug is available (for instance highly potent strains of cannabis, vaping liquids, and edible products) or in delivery systems for its consumption. In some jurisdictions, the legal market for recreational drugs has been accompanied by regulations that allow access to cannabis for medical and therapeutic purposes. Other important policy issues in this area include questions on what constitutes appropriate treatment for cannabis-related disorders, how to ensure policy synergies with tobacco control strategies, and what constitutes a harm reduction approach which is effective with this regard. The prevalence of cannabis use in Europe remains high in historical terms, and recent increases have been observed in some EU Member States. The potency of the drug, which has risen sharply in the last decade, also reaches high levels, whether it is cannabis resin or herbaceous cannabis. Beyond public health issues, there are concerns about what will be the impact of this large illicit market on community safety and how it might even be funding organized crime. Taking into account the several involved issues, defining what constitutes the most appropriate response to cannabis use is a task of growing complexity and importance [86,87].

## 5. Therapeutic Indications

The use of preparations derived from *Cannabis sativa* in medicine has a long history. However, this use has largely declined in the twentieth century, and its consumption for medical purposes was limited when cannabis was included in the United Nations Single Convention on Narcotic Drugs in 1961, and classified as not presenting known medical uses. Notwithstanding, there has been a reappearance of patient interest in using these drugs for the treatment of a variety of medical conditions, including chronic and cancer pain, depression, anxiety disorders, sleep disturbances and neurological disorders in the past 20 years, since their symptoms were reportedly improved by using cannabis.

This increased interest of patients in using cannabis medically was accompanied by a renewed interest of scientists on the potential medical use of several of the plant’s constituents. This occurred after the discovery in the early 1990s of a cannabinoid system in the human organism, which has been associated to the control of important biological functions, such as cognition, memory, pain, sleep and immune functioning. However, the early classification of cannabis as a drug with no medical use has made it difficult to adequately conduct clinical research on the matter. In the mid-1990s the medical use of cannabis for people with a variety of illnesses, such as chronic pain, terminal cancer and multiple sclerosis was legalized in several states of the USA, which was afterwards followed by many other states. In 1999, Canada introduced a program involving medical cannabis, which has expanded thereafter. In fact, since the early 2000s, several other countries have implemented the medical use of cannabis under specified conditions, for instance Israel (2001), the Netherlands (2003), Switzerland (2011), Czech Republic (2013), Chile (2015), Australia (2016), Norway (2016), Peru (2017), Germany (2017) and more recently Thailand (2018) among others legislated to allow the medical use of cannabis under specified conditions.

Most EU countries now allow, or are considering allowing, the medical use of cannabis or cannabinoids in some form [86,92]. “Sativex^®^”, containing approximately equal quantities of THC and CBD is the most recognized product marketed in a number of European countries. This product, which is administered by spraying inside the cheek or under the tongue, has been authorized in 17 EU Member States (Austria, Belgium, Czech Republic, Denmark, Finland, France, Germany, Ireland, Italy, Luxembourg, Netherlands, Poland, Portugal, Slovakia, Spain, Sweden, United Kingdom) and Norway for the treatment of muscle spasticity in patients with multiple sclerosis. Other products available in Europe are Marinol (Dronabinol), containing synthetic THC, which is used in cancer treatment, AIDS and multiple sclerosis; and Cesamet™ (Nabilone), containing a synthetic analog to THC, used for cancer treatment. However, national approaches vary widely in terms of both the products allowed and the regulatory frameworks governing their provision [92].

Other countries have however more restrictive laws, allowing only the use of certain cannabis-derived pharmaceutical products, such as Sativex^®^, Marinol or Epidiolex^®^ (CBD). In the United States, 33 states and the District of Columbia have legalized the medical use of cannabis, but at the federal level its use remains prohibited for all purposes [93,94]. In the following lines, a more comprehensive review will be made on the uses of cannabis to treat medical conditions. This search was made based on the PubMed and Google Scholar databases using the following search strings: “cannabis insomnia”; “cannabis and anxiety”; “cannabis and post-traumatic stress disorder”; “cannabis and fibromyalgia”; “cannabis and pain management”; “cannabis and appetite treatment”, “cannabinoids and dermatology or skin therapy”, “cannabinoids and glaucoma”, “cannabinoids and infections therapy”, “cannabis and epilepsy”, “cannabis and Tourette syndrome”, “cannabis and Parkinson disease”, “cannabis and Alzheimer disease”, “cannabis and multiple sclerosis”, “ cannabis and nausea and/or vomiting”.

This section summarizes the evidences on the properties of cannabis and cannabinoids from systematic reviews of random controlled clinical trials. A particular challenge in interpreting the evidences is that several different cannabis products and preparations have often been used and may have contained several different active ingredients. 

### 5.1. Pain Management 

Pain management is a problem of general interest and a public health issue, which increasingly attracts the attention of several countries that consider the use of cannabis in cases of acute or chronic pain. It can be said that legal and political decisions influence the habits of cannabis use in these situations. Since the early 2000s, several studies have been published on the advantages and limitations of cannabis implementation in the therapy of patients with persistent pain. In recent years there has been an increase in the number of studies and reviews conducted in this area.

As an example, the study by Bigand et al. [95] aimed at analyzing health outcomes of adults using opioids for pain with the addition of cannabis use, evaluating the beneficial or non-beneficial effects of this. One-hundred-and-fifty patients aged between 19 and 85 years prescribed with opiates for the treatment of persistent pain, including pain correlated to cancer, were included in this study. The management of physiological symptoms was considered by patients to be a major benefit of cannabis use, and most reported the relieve of symptoms, namely pain, insomnia and nausea, anxiety, depression, and stress. Negative symptoms such as weight gain, red eyes, dry mouth, nausea, fast heart, lack of concentration, poor memory, drowsiness, apathy, lack of motivation, and more serious symptoms such as increased anxiety, paranoia, seizures and anaphylaxis due to allergic reaction were reported as well. The authors of this study concluded that legalizing cannabis for medical and recreational use brings more knowledge to both patients and health professionals who become more aware of the effects of cannabis on pain-related symptoms, thus enabling open communication, more treatment options, and greater safety for patients. 

By the end of 2017, Armour et al. [96] conducted an online study of endometriosis in the female population of Australia between the ages of 18 and 45. Current medical treatments do not generally provide sufficient pain relief or have intolerable side effects, and this research aimed to determine the prevalence of use of common forms of self-management. Four hundred and eighty-four responses were considered as valid. The authors concluded that women using cannabis and hemp/CBD oil reported these substances among the most well-evaluated in terms of effectiveness in reducing pain. However, as the number of women using it was small and the results were self-reported, clinical trials are needed in this area to determine any possible role of legally-obtained medicinal cannabis in the management of endometriosis.

The pain of rheumatic patients is one of the reasons to consider using medicinal cannabis as an alternative treatment to the implemented therapies, such as opioids. Fitzcharles et al. [97] compiled the Canadian legislation and the studies conducted on the effects of medicinal cannabis in these patients, and have concluded that patients should be provided with health professionals that have the best evidence-based information on the beneficial effects as well as on cannabis damage, facilitating dialogue between patients and physicians, in an attempt to reduce drug-related harms, not only to patients but also to society. However, clinical trials of medicinal cannabis in these patients have not yet been conducted, and the beneficial evidence for pharmaceutical cannabinoids in fibromyalgia, osteoarthritis, rheumatoid arthritis, and back pain are insufficient. On the other hand, there is evidence of a high risk of harm. They have also concluded that medical cannabis can provide relief for some patients and short-term risks such as psychomotor effects, appetite changes, dizziness, mood effects, and more serious effects such as disorientation and psychosis may even be anticipated. It is also known that cannabis should not be smoked because the inhalation of combustible products carries a bronchial risk; however, long-term risks have not yet been determined.

Palace et al. [98] developed a state program with safe and controlled administration of medical cannabis to resident patients diagnosed with chronic pain, neuropathy or Parkinson’s disease. These authors concluded that the elderly who used medicinal cannabis demonstrated significant decreases in the use of prescription drugs, mainly the use of opioids, and medicinal cannabis should be seen as an additional clinical option to relief the symptoms. As in the previously described studies, they concluded that with the acceptance and diffusion of this knowledge, more health professionals could help patients to benefit and request this alternative therapy. These authors stated that the available medicinal cannabis formulations contained a standardized dose of CBD with little or no THC; this is capable of eliminating psychoactive effects in the elderly, improving the safety profile in chronic pain treatment.

Shin et al. [99] have reviewed the available literature on the use of cannabis and cannabinoids in the treatment of cancer pain, such as breast cancer, lymphoma, and cervical cancer. As the number of studies on the efficacy of these compounds is limited, it becomes challenging and difficult to give a definite recommendation for a certain practice. Although evidence is scarce, they is enough to justify more research on the use of cannabis in cancer, and this process is facilitated in cases of legislation changes concerning its consumption. Limitations such as the small number of patients in the study, abstinence, withdrawal due to negative side effects, and use of different pain scales were found. The authors concluded that some of the studies reported that THC is more effective than the placebo, but other studies have found THC to be no more effective than placebo, making it difficult to find agreement on its use for treatment.

More recently, Campbell et al. [100] have delineated the currently available evidence on cannabis and medicinal cannabis for the treatment of non-oncologic chronic pain, such as neuropathic pain, multiple sclerosis-related pain and visceral pain, which are among the most important reasons for research in the use of these compounds. These authors concluded that there are several limitations that need to be studied because the patients’ perceptions regarding the efficacy of cannabinoids in cases of pain are not included in the existing evidence. This makes it important to manage patients’ expectations and to better understand what the potential side effects are, as these can limit the drug’s use. It is accepted that in most of these studies a combined dose of THC and CBD was used, with little proof of the benefits that other cannabis-based medicines have in neuropathic pain.

Noteworthy is the work developed by Perron et al. [101], who intended to describe abstinence patterns of cannabis in a large sample of patients who used medical cannabis, in order to test the association between withdrawal symptoms and functioning. Participants in this study were adults over 21 years of age interested in having or maintaining medical certification for the use of cannabis for the treatment of chronic pain. Two-thirds of the patients reported at least one symptom of moderate or severe abstinence and the most frequently observed symptom was difficult sleep, which was followed by anxiety, irritability and appetite disturbances. Abstinence symptoms occurred at significantly higher rates for patients with poor mental functioning compared to patients with high mental functioning. In addition, there was no visible association between physical functioning and withdrawal symptoms. The authors concluded that these abstinence symptoms were highly prevalent in patients who used medical cannabis at least three times a week. These findings, although limited, may be important for patients and may assist health professionals in deciding which therapies to apply, and therefore a more effective intervention, as they become better aware of the negative consequences of using medical cannabis. Additional studies will always be necessary, addressing other issues, such as legislation and the differences between countries and how patients may be affected by this.

Cannabinoids may also be used in the treatment of fibromyalgia, namely in the relief of lumbar pain, fatigue and mood disturbances, symptoms which are usually associated to this disease [102,103]. Some researchers have found a correlation between deficiencies in endocannabinoid system and fibromyalgia, and recently published studies have focused on the therapeutic effects of cannabis on it [97,104,105,106]. Yassin et al. [107] evaluated the improvement of pain and function in situations of patients with fibromyalgia, with the addition of cannabis therapy to the standardized pharmacological analgesic treatment. A cross-over observational study was conducted in which thirty-one patients between 21 and 75 years were observed and treated with the standard therapy (oxycodone hydrochloride, naloxone hydrochloride, and duloxetine) for three months. After that time, patients were able to choose cannabis treatment for six months. The authors concluded that the standardized therapy led to a small improvement when compared to non-treated situations, while the cannabis treatment allowed a significant improvement after three months of implementation and that this improvement was maintained at six months. There was, thus, an advantage of cannabis treatment compared to the medication normally prescribed in a patient with low back pain associated to fibromyalgia. No patient had to discontinue therapy due to adverse events, and most of them have decreased or discontinued standard analgesics consumption. The authors further concluded that additional randomized clinical trials are needed to assess whether the results can be generalized to the general population, as the mechanism of cannabis pain relief associated to this pathology is not defined. Another associated problem is the lack of standardization of the amount of THC/CBD that should be applied. The proportion of THC to CBD recommended in the chronic pain therapeutic in this trial was 1:4.

Pain treatment is undoubtedly the most studied therapeutic indication of cannabis. In general, researchers have found that cannabis-based medicines are probably effective for treating the neuropathic pain and painful spasms usually associated to multiple sclerosis [108], as well as neuropathic pain associated to diabetes, HIV, and other illnesses. However, it is unclear whether or not smoked marijuana is effective in reducing pain in multiple sclerosis [108,109]. Cannabis can be used to treat chronic pain associated to cancer and other causes, and studies generally suggested cannabis-related improvements in chronic pain measures in cancer patients [103,110,111]. Most studies used Sativex^®^ preparations, and have generally shown positive results [111,112].

### 5.2. Epilepsy

Epilepsy is a disorder from the CNS in which the brain activity becomes abnormal, causing episodes of involuntary movement that may involve a part or the entire body. Globally, an estimated 2.4 million people are diagnosed with epilepsy each year and has an annual cumulative incidence of 67.77 per 100,000 persons [113]. 

Drug resistance epilepsy is defined as failure to stop all seizures in a patient who had adequate trials of at least two appropriate medications, such as Dravet syndrome and Lennox–Gastaut syndrome, in which it is usual to have both generalized and focal drug-resistant seizures [114].

New medications have been approved in the past two decades, but these have not reduced the proportion of patients with intractable epilepsy [115]. In the last few years enormous interest has been generated by social and news media about the beneficial effects of cannabis products for the treatment of drug-resistant or refractory epilepsy. This is the most recent therapeutic approach for cannabis products, and evidences found in studies using animals will be separated from those obtained in clinical trials aiming at a better systematization of the gathered information. 

#### 5.2.1. Evidence in Animal Models and Basic Pharmacological Mechanisms

After centuries of anecdotal reports, there has been an expansion of preclinical trials in order to investigate the pharmacologic potential of various phytocannabinoids as anticonvulsant drugs. These reports evaluate those cannabinoids that do not have psychoactive properties, mainly CBD but also including cannabidivarin and Δ9-tetrahydrocannabivarin.

In the early 1970s, CBD was found to have anticonvulsant properties in experimental animal models. Carlini et al. [116] suggested that 200 mg/kg of CBD significantly protected the mice from the convulsant and lethal effects of leptazol. Furthermore, Consroe et al. [117] suggested that the CBD effects were comparable to those of phenytoin and enhance the anticonvulsant effects of phenobarbital and phenytoin. In subsequent reports, CBD consistently showed anticonvulsant effects in several animal epileptic models including: maximal electroshock test (mES) [118,119,120]; 6 Hz and subcutaneous metrazol threshold test [120]; pentylenetetrazol [121]; pilocarpine and penicillin models [122].

To date, the antiepileptic mechanism of action of CBD remains unknown. CBD has low affinity for both CB1 and CB2 endocannabinoid receptors and is therefore likely to be exerting its activity via cannabinoid receptor-independent [119,121,123]. In contrast to CB1, CB2 receptors are attractive targets for the development of novel therapeutic approaches, and nowadays it is acknowledged that the anti-inflammatory properties of cannabinoid agonists also involve these last receptors. CB2 receptor activation has proven to decrease the production of proinflammatory molecules in a number of neural cell types, such as rat microglial cells, primary mouse astrocytes, human microglial, and THP-1 cells [124,125,126,127]. Other potential mechanisms includes agonist at transient receptor potential (TRP) cation channels (specifically calcium channel modulation) [128]; blocks T-type calcium channels [129]; modulation of serotonin (5-HT_1A_/5-HT_2A_) and adenosine (A_1_ and A_2_) receptors [130,131] and modulation of voltage dependent anion-selective channel protein 1 (VDAC1) [132].

Cannabidivarin (propyl analogue of CBD) is an effective anticonvulsant in a broad range of seizure models [133]. Like CBD, cannabidivarin anticonvulsant properties are not mediated by CB1 receptor and have agonist effects at TRP cation channel [134]. Δ9-tetrahydrocannabivarin, another cannabinoid found in cannabis, exerts some anti-epileptiform effects in vitro and very limited anticonvulsant effects on pentylenetetrazole–induced seizure model. The anticonvulsant properties are consistent with a CB1 receptor–mediated mechanism [135].

Although cannabis has been used medicinally for centuries, it is only within the last few decades that there is accumulated evidence that some cannabinoids have anticonvulsant properties in animal epileptic models. Despite this evidence, the mechanisms by which these compounds exert anti-seizure effects are poorly understood. Identification of mechanisms underlying the anticonvulsant efficacy of cannabinoids is critical to determine other potential treatment options.

#### 5.2.2. Clinical Evidence in Epilepsy

From the 1980s to the last few years, several studies have been conducted, some of them randomized and blinded, that involve the use of isolated CBD as a therapeutic option for epilepsy.

Mechoulam and Carlini [136] treated four patients with CBD (200 mg daily) and five patients with placebo. CBD treatment showed that 75% of the patients had a remarkable improvement in seizures throughout the entire three-month period while none of the placebo patients showed any improvement.

Cunha et al. [137] conducted a trial of fifteen adult patients with focal-onset epilepsies. The patients were given between 200–300 mg of CBD or placebo. Patients treated with CBD, 7/8 reported improvement in seizures; while seven of the patients who received placebo remained unchanged [137].

In contrast, two studies provided a few details (patients with uncontrolled seizures were randomized into either placebo or CBD groups) but suggested little or no difference in seizure frequency between placebo and CBD groups. In Ames 1985, 12 patients institutionalized due to mental retardation with uncontrolled seizures were given three capsules of placebo or 100 mg of CBD [138]. The other trial was an unpublished abstract from a conference. Twelve patients were treated with a single-blind placebo for six months followed by double-blind 300 mg of CBD or placebo in a cross-over trial lasting an additional 12 months [138].

Insufficient preclinical and clinical data have intersected with a need for more effective therapies for drug resistance epilepsy, which created a demand for CBD-based treatments. Recently, there has been a surge in clinical trials investigating the additive effects of highly purified CBD treatment (Epidiolex^®^; GW Pharmaceuticals) to daily antiepileptic drugs (AEDs) regimens in both children and adults [139,140,141,142,143,144].

In an open-label interventional trial, 214 patients (aged 1–30) with severe, intractable, childhood-onset, treatment resistant epilepsy, were given oral CBD at 2–5 mg/kg/day, then up-titrated until intolerance or to a maximum dose (25 or 50 mg/kg/day) for 12 weeks. Add-on treatment with CBD led to a clinically meaningful reduction in seizure frequency and had an adequate tolerability and safety profile [141].

Devinsky et al. [142] reported results on both efficacy and safety data on patients who received Epidiolex^®^ as part of their daily regimen for at least 14 weeks. In this double-blind, placebo-controlled trial, 120 children and young adults with drug-resistant Dravet syndrome, were randomly assigned in a 1:1 ratio to receive CBD oral solution (dose escalated up to 20 mg/kg/day) or placebo. This trial showed that CBD resulted in a greater reduction in convulsive–seizure frequency than placebo. The median frequency of convulsive seizures per month decreased from 12.4 to 5.9 with CBD, in comparison with a decrease from 14.9 to 14.1 with placebo. At least a 50% reduction in convulsive seizure frequency was 43% with CBD (27% with placebo) and 5% of patients became seizure-free (0% with placebo) [142].

In 2018, a series of trials showed the long-term safety and efficacy of CBD in children and adults with treatment-resistant epilepsies. An update on an expanded access program provided the safety outcomes up to 144 weeks and efficacy up to 96 weeks in more than 600 patients. Results from this ongoing expanded access program supported that add-on CBD may be an efficacious long-term treatment option for treatment-resistant epilepsies. CBD treatment was associated with 51% and 48% reductions in median monthly convulsive and total seizures respectively after 12 weeks. Reductions were similar among visit windows through 96 weeks of treatment and CBD was generally well tolerated [139].

In addition, Devinsky et al. [143] presented an interim analysis of the safety and efficacy from 264 patients with Dravet syndrome treated with long-term CBD (mean modal dose 21 mg/kg/day, median treatment 274 days). In this open-label extension trial, CBD treatment had an acceptable safety profile. Sustained reductions in convulsive and total seizures were observed through 48 weeks and 85% of patientscaregivers reported improvements in overall condition [143].

In two additional randomized, double-blind, placebo-controlled trials, CBD treatment at a dose of 10 mg or 20 mg per kilogram per day was associated with greater reductions in the frequencies of seizures among children and adults with the Lennox–Gastaut syndrome [140,144].

Adverse events that occurred more frequently in the CBD treatment with the concomitant AEDs included diarrhea, vomiting, fatigue, pyrexia, somnolence, and abnormal results on liver-function tests [139,140,141,142,143,144]. 

Some effects of CBD may relate to interactions with other AED [145]. CBD inhibits cytochrome P450 (CYP2C19) and produces an increase in plasma concentrations of N-desmethylclobazam (active metabolite) [146]. In patients taking clobazam and CBD who experience bothersome sedation, a reduction of the clobazam (or CBD) dose may be considered. 

In these trials [139,140,141,142,143,144], abnormal liver function test results were noted in participants taking concomitant valproate, suggesting a CBD-valproate interaction. CBD had no effect on systemic levels of valproate, which suggests that the interaction may be pharmacodynamics [147].

Based on the results obtained in these clinical trials, in June 2018, the US Food and Drug Administration (FDA) approved Epidiolex^®^; for the treatment of seizures associated with Lennox–Gastaut syndrome or Dravet syndrome in patients two years of age or older.

Recently, there has been a growing interest in using of CBD-enriched cannabis oil (medical cannabis) for the treatment of drug-resistance epilepsy. One case (called Charlotte case) that received a lot of media attention, reported a little girl who showed substantial decreased seizures when she started therapy with a high concentration of CBD/THC cannabis oil. Charlotte was given a low dose of a sublingual preparation of a cannabis extract and slowly increased the extract dose, keeping the THC content sufficiently low to avoid psychotropic effects. For the first time, Charlotte experienced seven consecutive days without a single seizure. With a baseline frequency of 300 convulsions per week, Charlotte had a >90% reduction in seizures [148].

Several online forum surveys have been performed examining the effects of medical cannabis for intractable pediatric epilepsy. In a telephone/Internet survey, 84% of parents who had administered CBD-enriched cannabis to 19 children with epilepsy (Dravet syndrome, Doose syndrome, Lennox–Gastaut syndrome and idiopathic epilepsy) reported substantial reductions in seizures frequency [149].

Another online parental survey focused on perceived efficacy, dosage, and tolerability of medical cannabis in 117 children with epilepsy syndrome (infantile spasms, Lennox–Gastaut syndrome, and other). The perceived efficacy and tolerability were similar across etiologic subgroups, with 85% of them reporting some reduction in seizure frequency and 14% reporting complete seizure freedom. The median duration and the median dosage of CBD exposure were 6.8 months and 4.3 mg/kg/day, respectively [150].

Finally, Press et al. [151] presented a retrospective review of children and adolescents (*n* = 75) with various epileptic encephalopathies who were given oral cannabis extracts. Thirteen percent of patients reported to have a reduction in seizure frequency (>50% in response). The responder rate varied based on epilepsy syndrome: Dravet 23%, Doose 0%, and Lennox–Gastaut syndrome 88.9% [151].

Other beneficial effects reported in these three studies included increased alertness, better mood, and improved sleep [149,150,151]. In addition, Hussain et al. [150] reported improvements in language and motor skills when using CBD. The few side effects reported included increased appetite, somnolence/fatigue, and an increase in seizure frequency [149,150,151].

The limitations of the online surveys are of paramount importance and introduce the possibility for numerous sources of confounding: bias selection patients, lack of placebo controls, unblinded self-assessment of efficacy/tolerability, inconsistency of CBD concentration and inaccuracy in the identification of patients’ epilepsy syndromes.

Three publications on clinical trials appeared between 2016 and 2018 evaluating the efficacy and safety of medical cannabis for the treatment of refractory epilepsy. An observational longitudinal study suggested that adding CBD-enriched cannabis extract to the treatment regimen of patients with refractory epilepsy may result in a significant reduction in seizure frequency according to parental reports [152]. Fifty-seven children and adolescents (1–20 years) with epilepsy of various etiologies were treated with cannabis oil extract (CBD/THC ratio of 20:1) for at least three months.A dose of 2–5 mg/kg/day (divided into three daily doses) was added to the baseline antiepileptic regimen and the dosage was incremented until intolerance (THC did not exceed 0.15–1.35 mg/kg/day). Of the 46 patients included in the efficacy analysis, 43.5% had a seizure reduction >50%; 22% had a reduction of 50–75%; 30% had a reduction of 75–99%; and 4% were seizure-free [152].

The second report was a prospective open-label trial that described safe dose, tolerability, and efficacy of a cannabis oil containing CBD/THC ratio of 50:1 in children with Dravet syndrome. Nineteen participants completed the 20-week intervention. Mean dose achieved was 13.3 mg/kg/day of CBD (range 7–16 mg/kg/day) and 0.27 mg/kg/day of THC (range 0.14–0.32 mg/kg/day). Cannabis oil treatment resulted in a significant reduction in motor seizures of 70.6%, improvement in quality of life and reduction in electroencephalogram spike activity [153].

The last report was a retrospective study describing the effect of medicinal cannabis on children and adolescents with intractable epilepsy. Seventy-four patients started the treatment (CBD/THC ratio of 20:1) for at least three months (average six months). CBD dose ranged from 1 to 20 mg/kg/day and THC dosage did not exceed 0.5 mg/kg/day. Most of the patients (89%) reported a reduction in seizure frequency (18% reported 75–100% reduction; 34% reported 50–75% reduction, 12% reported 25–50% reduction, and 26% reported <25% reduction) [154].

CBD-enriched medical cannabis is shaping up to be a very promising anticonvulsant option, with favorable safety profile. For a clearer judgment of the potential therapeutic effects, the risks and legality of a cannabis oil, it is important to know its exact composition. Consistent formulation through strict methodology will allow to assess the synergism of other phytocannabinoids as well as other compounds such as flavonoids and terpenoids.

### 5.3. Neurodegenerative Disorders: Parkinson’s Disease, Alzheimer’s Disease, and Multiple Sclerosis

#### 5.3.1. Parkinson’s Disease

Parkinson’s Disease (PD) is a progressive neurodegenerative disorder characterized clinically by symptoms such as bradykinesia, rigidity, tremor, and postural instability. These symptoms are produced by brain dopaminergic denervation at the striatum level and progressive death of dopaminergic neurons in the pars compacta of the substantia nigra. Parkinson’s Disease is multifaceted with disparate etiologies, a range of clinical symptoms and variations in pathology [155]. Scientific evidence indicates that immunological pathways are important in the pathophysiology of PD [156].

The impact cannabis has on motor and nonmotor symptoms of PD may be modulated by the dopaminergic, serotonergic, adrenergic, and neuroprotective properties of cannabinoids. The CB1 receptor is one of the most abundant receptors in the CNS. The CB1 receptor is highly expressed in the basal ganglia, the brain structures primarily affected in PD [157]. This specific localization of CB1 may explain the effect of cannabinoids on cognitive and motor activity. The CB2 receptors are expressed primarily in peripheral immunocompetent cells and lymphoid organs but are also expressed in the CNS [158]. The CB2 receptors have been found to modulate microglia activation and may play a role in neuroinflammation/neuroprotection [159], since as mentioned above, their activation has proven to decrease the production of proinflammatory molecules in a number of neural cell types. 

Furthermore, pre-clinical research demonstrated that cannabinoids prevent neuronal damage into the nigra pars compacta in rodents probably by its antioxidant activity, possibly associated a CB receptor-independent [160]. In an additional study Peres et al. [161] reported that CBD’s antioxidant and anti-inflammatory actions would attenuate reserpine-induced motor and cognitive impairments in an experimental model.

In animal models of PD, the levels of CB1 receptors appear to be downregulated in the substantia nigra and the globus pallidus at early phases (12 months of age). By contrast, CB1 receptors showed an elevation in the same areas when animals were analyzed at older ages [162]. Together, these studies suggest a complex link between the pathophysiology of PD and changes in the endocannabinoid system.

The results of clinical trials examining the role of cannabinoids in the treatment of PD are mixed. A first preliminary clinical open pilot study reported that CBD treatment (100–600 mg/day CBD in capsules with sesame oil vehicle) for six weeks in PD patients, improved dystonia in all patients (*n* = 5) in a range from 20 to 50%. However, in two patients CBD treatment at doses over 300 mg/day exacerbated the hypokinesia and resting tremor. Side effects included hypotension, dry mouth, psychomotor slowing, lightheadedness, and sedation [163].

An open-label pilot study reported that CBD in flexible dose (started with an oral dose of 150 mg/day) for 4 weeks in six patients with psychosis in PD, improved the psychotic symptoms [164].

In another trial, 21 PD patients were randomized to placebo, CBD 75mg/day or CBD 300 mg/day for six weeks. No significant changes were found between CBD and placebo in scores obtained with Unified PD Rating Scale (motor and general symptoms score) and possible neuroprotective effects. However, in the PD Questionnaire (well-being and quality of life) significant differences were found between the total score of the placebo and CBD 300 mg/day groups. No serious adverse events were reported [165].

Many studies examining the efficacy of cannabis in PD are limited to questionnaires and observation in patients actively consuming marijuana either recreational or prescribed. These observational/questionnaires uncontrolled studies suggest that cannabis could improve motor symptoms. In some studies, patients who consumed cannabis reported improvements in some of the symptoms of the PD: tremor, bradykinesia, rigidity, problems with sleep and pain [166,167,168,169,170]. In other study, five patients with idiopathic PD found no benefit for tremor following a single administration of smoked cannabis (1 g. cigarette containing 2.9 % THC) [171].

Unfortunately, a few randomized double-blind clinical trials have been carried out with cannabis on people with PD. A crossover study in 19 PD patients demonstrated that an oral cannabis extract (1.25 mg CBD and 2.5 mg THC and per capsule, maximum daily dose 0.25 mg/kg THC) was well tolerated and had no pro- or antiparkinsonian action [172].

At present, a phase II randomized, open-label, double-blind trial, is underway to evaluate the tolerability, safety and dose-finding of cannabis oil for pain in PD. Mixed cannabis oil preparation consisting of three differing formulations of THC and CBD (proportions of THC and CBD in the following ratios: 18:0.2; 10:10; 1:20) will be administered to a total of 15 assigned patients randomly to one of the three groups. Estimated primary completion date will be available in December 2019 [173].

#### 5.3.2. Alzheimer’s Disease

A widely accepted theory underlying the pathophysiology of Alzheimer’s disease (AD) is the deposition of amyloid-β protein in specific brain regions leading to localized neuroinflammatory responses and accumulation of intra-cellular neurofibrillary tangles. These events result in neuronal cell death with accompanying loss of functional synapses and changes in neurotransmitter levels [174].

Pre-clinical studies suggest that the endocannabinoid system protects against neuronal cell death, oxidative stress, and inflammation, events associated with the development of AD. In-vitro experiments determined that THC could bind and competitively inhibit the enzyme acetylcholinesterase as well as prevent acetylcholinesterase-induced amyloid-β protein aggregation [175]. Several mechanisms have been suggested to explain CBD neuroprotection: reduction of oxidative stress and anti-apoptotic effects [176], inhibition Aβ-induced tau protein hyperphosphorylation which leads to the formation of neurofibrillary tangles [177], decrease in amyloid-β production and amyloid-β induced neurodegeneration by inhibition of inducible nitric oxide synthase (iNOS) and interleukin-1β protein expression [178].

Long-term CBD treatment (CBD 20 mg/kg/day for 8 months) in a transgenic model of AD, prevented social recognition deficit (not associated with any changes in amyloid load or oxidative damage). This study revealed a subtle impact of CBD on neuroinflammation, cholesterol and dietary phytosterol retention [179].

Clinical evidence indicates that dronabinol (synthetic form of THC) and medical cannabis may have some benefit in treatment of behavioral and psychological symptoms of dementia. 

There were two open-label prospective studies [180,181], two randomized double-blind placebo-controlled repeated crossover trials [182,183], a double-blind placebo-controlled crossover design [184], a placebo-controlled trial [185] and one retrospective study [186]. Five reports used dronabinol [180,182,184,185,186] and two reports used medical cannabis [181,183]. Three studies were 2 weeks in duration [180,182,185], one trial was 17 (mean) days long [186], another study was 4 weeks in duration [181] and two trials were 12 weeks in duration [183,184]. 

The dose of dronabinol were 2.5 mg/day [180,182,184,185] and one study used dronabinol at 7.03 mg/ day (mean) [186], one trial used THC up to 3 mg/day [183], and one used maximal dose of 15 mg/day [181].

Six studies indicated that symptoms improved with the use of cannabinoids: reduction of nocturnal motor activity and agitation [180,182,185], decreased of delusions, agitation/aggression, irritability, apathy, sleep and caregiver distress [181,184], decreased of aberrant vocalization, motor agitation, aggressiveness and resisting care [186]. Only one found that there was no benefit to using THC when compared with placebo over a 12-week period at a maximum dose of 3 mg/day [183]. Adverse effects reported with dronabinol were tiredness, sedation, somnolence, confusion, and euphoria for a longer period [184,186].

The limitations of these studies include a small number of participants, short study duration and lack of placebo group. Current data should be considered as preliminary. It would be premature to say that the cannabinoids have any effect on dementia symptoms or progression. Additional double blinded, randomized, placebo-controlled trials are needed to evaluate the efficacy and safety of cannabinoids in AD. In addition, CBD has demonstrated effects in pre-clinical AD models suggesting a deeper investigation to clarify the potential clinical utility of CBD.

#### 5.3.3. Multiple Sclerosis

Multiple sclerosis is a chronic autoimmune, inflammatory neurological disease characterized by demyelination in the CNS caused by inflammatory immune-mediated attacks [187,188]. In 2015 there were more than 2 million people worldwide affected by multiple sclerosis [189] and it is currently incurable [190].

Typical syndromes at presentation include monocular visual loss due to optic neuritis, limb weakness or sensory loss, double vision or ataxia [191]. A progressive clinical course develops in many of the persons affected, eventually leading to impaired mobility and cognition.

Pre-clinical studies suggest that cannabis and individual cannabinoids improve the signs of motor dysfunction in experimental models of multiple sclerosis [192].

Lyman was one of the first to report the effects of THC in animals with autoimmune encephalomyelitis (multiple sclerosis model). In that study, affected animals treated with THC either had no clinical signs of the disorder or showed mild clinical signs with delayed onset. In addition, examination of central nervous system tissue revealed a marked reduction of inflammation [193].

Additional reports have supported and extended these findings demonstrating that THC, but not CBD, ameliorated both tremor and spasticity and reduced the overall clinical severity of the disease [194,195].

Some reports highlight the importance of the CB1 receptor in controlling spasticity, tremor and the neuroinflammatory response in multiple sclerosis [195,196,197]. Although great evidence suggests cannabinoids exert immunosuppressive effects, it is believed that the neuroprotective properties of cannabinoids may be more relevant than their immunosuppressive characteristics in multiple sclerosis [197,198,199].

Cannabinoids therapeutic potential has aroused considerable interest in multiple sclerosis treatment. Patients with multiple sclerosis are using or considering using cannabis for a range of symptoms. Recent studies have indicated that there is a wide acceptance of cannabis within the multiple sclerosis community, with 20–60% of MS currently using cannabis, and 50–90% would consider usage if it were legal and more scientific evidence was available [200,201]. 

In some countries a mixture of cannabinoids (Nabiximols/Sativex^®^: mixture of THC and CBD in an approximate ratio of 1:1) has been approved for the symptomatic treatment of multiple sclerosis spasticity and neuropathic pain in cases in which previous medication has proved ineffective [202].

Recently, Torres-Moreno et al. [203] conducted a systematic review and meta-analysis to assess the efficacy and tolerability of medicinal cannabinoids by oral or oromucosal administration in the symptomatic treatment (spasticity, pain, and bladder dysfunction) of patients with multiple sclerosis. The medicinal cannabinoids evaluated were nabiximols; oral cannabis extract contains THC and CBD; dronabinol and nabilone. 

Nineteen studies involving 3161 patients were identified to have satisfied the eligible criteria (randomized, placebo-controlled, double-blind, and parallel/crossover-designed trials for a minimum length of treatment of 2 weeks). Medicinal cannabinoids produce a limited and mild reduction of subjective spasticity, pain, and bladder dysfunction in patients with multiple sclerosis, but no changes in objectively measured spasticity. In the analysis of subjective spasticity, significant differences were observed with respect to the active treatments of oral cannabis extract and nabiximols. Efficacy in pain of oral cannabis extract and nabilone was also demonstrated, in addition to efficacy in bladder dysfunction for oral cannabis extract. 

Variability in the compounds studied also influences the interpretation of tolerability results. In the total adverse events analysis, there was a higher risk of adverse events in nabiximols treatments and a higher risk of withdrawals due to adverse events in nabiximols; oral cannabis extract and dronabinol treatment.

Currently, there is no evidence of reports that evaluate the efficacy of cannabinoids versus other treatments (corticosteroids, anticholinergic agents) in multiple sclerosis. In addition, research into the possible combinations might bring about greater synergy benefits than in an individual form.

Future research should also be aimed at obtaining more conclusive evidence about the efficacy of cannabis or individual cannabinoids against the signs and symptoms of multiple sclerosis, dose and route of administration and at exploring strategies that maximize separation between the beneficial therapeutic effects and the undesirable effects.

### 5.4. Post-Traumatic Stress Disorder

In the present day, clinicians need to counsel patients with post-traumatic stress disorder (PTSD) who are using or requesting to use cannabis for therapeutic or recreational purposes. For this reason, it is important to understand benefits or potential harms of cannabis in this disorder [204]. O’Neil et al. [205] report that in a general way, there is insufficient evidence regarding the benefits and harms of cannabis preparations for patients with PTSD. A review made by these authors points several trials treating PTSD patients with THC and CBD, randomizing its amounts present in smoked cannabis. The outcomes observed were categorized as changes in the Clinician-Administered PTSD Scale.

However, according to Bitencourt and Takahashi [206], human and animal studies suggest that CBD may offer therapeutic benefits for PTSD, revealing fewer side effects than the pharmacological therapy currently adopted for this disorder. Although with evidence that points to the modulation of the endocannabinoid system, the authors recognize that more studies should be performed for a better understanding of the neurobiological mechanisms. CBD was also studied by Elms et al. [207] who observed that patients taking daily oral CBD over an 8-week period demonstrated an overall decrease in PTSD symptom severity. The authors recognize the good toleration of CBD and that only a minority of patients did report fatigue and gastrointestinal discomfort. Steenkamp et al. [208] report that their clinical and preclinical studies suggest the cannabinoids may offer therapeutic benefits in this disorder, and that the pharmacological enhancement of endocannabinoid system signaling has yielded promising results in rodents, preventing dysfunctional stress-related processes. The authors also recommend future controlled studies in humans for a better evaluation of the relation between PTSD and cannabis.

### 5.5. Tourette’s Syndrome

Tourette’s syndrome (TS) is an inherited neuropsychiatric disorder characterized by the presence of multiple motor tics and at least one vocal tic [209]. Although the cause of TS is unknown, current research points to abnormalities in certain brain regions (basal ganglia, frontal lobes, and cortex), the circuits that interconnect these regions, and the neurotransmitters dopamine, serotonin, and norepinephrine [210,211]. 

Since the central endocannabinoid system is an important modulatory in the brain that influences and controls all important neurotransmitter systems, it can be speculated that TS might be caused by a dysfunction in the central endocannabinoid system. Noteworthy, there is a strong interaction between the dopaminergic and the central endocannabinoid system [212,213], particularly in basal ganglia [214,215]. Therefore, it can also be speculated that cannabinoids may inhibit dopaminergic activity in brain areas associated with motor control resulting in movements such as tics [216].

For many patients with TS, available medications do not help with their symptoms, or cause significant side effects. Cannabinoids have been explored as a treatment for TS since the 1980s. In 1988, for the first time it was suggested that cannabis might be such an alternative treatment option for TS. Sandyk et al. [217] reported three patients who experienced a reduction in motor tics and obsessive-compulsive behavior when smoking cannabis. No side effects occurred, and treatment effect was stable over time and did not decrease [217]. Subsequently, a small number of case studies has been published describing beneficial effects of cannabis in patients with TS. In most of these cases, the authors report about beneficial effects on both tics and psychiatric symptoms and no reports available about severe side effects [218,219].

Later, two clinical trials investigated the effect of oral THC in TS patients. In a double-blind placebo-controlled trial 12 adult patients were randomly treated with oral THC (gelatin capsules at 2.5 and 5.0 mg) or placebo [220]. Patients received a single dose of THC (5; 7.5 or 10 mg) according to their body weight, sex, age, and prior use of cannabis. Motor and vocal tics and obsessive-compulsive behavior were improved in the treatment groups. No side effect or mild transient adverse reaction (headache, nauseas, ataxia, and anxiety) were reported in patients who had received 7.5 or 10 mg of THC [220]. The second report is a randomized, double-blind, placebo-controlled study with 17 patients with TS treated over a 6-week period with up to 10 mg/day of THC [221]. Tic severity and global clinical outcome scores were improved in some patients, while other patients did not benefit from THC treatment. In addition, THC reached effectiveness after a three-week period treatment, which persisted or even increased after more than four weeks. No serious side effects occurred during the study [221]. 

While these trials have shown promising results, recently Abi-Jaoude et al. [222] conducted a retrospective study of cannabis (smoked or vaporized) effectiveness and tolerability in 19 adult patients with TS. Nine patients had previously participated in trials with one or more pharmaceutical cannabinoid. The frequency of use varied significantly, from frequent usage of small doses throughout the day to daily use for one week followed by three weeks off. The estimated average total daily dose also varied substantially, from less than 0.1–10 g, for a median of 1 g daily. Tic severity decreased by 60% and 94.7% of the patients were at least “very much improved” or “much improved” according to the clinical global impression severity scales. Cannabis was generally well-tolerated, although most participants reported side effects. It is interesting that patients reported to have much greater improvement in their symptoms using inhaled cannabis than using cannabinoid pharmaceuticals (pure oral THC, THC/CBD oromucosal spray, or the oral cannabinoid nabilone) [222].

To date, a double-blind, randomized, crossover pilot trial is underway to assess the efficacy and safety of three vaporized medical cannabis products with different THC and CBD contents, as well as placebo, in adults with TS [223]. Estimated completion date will be available on May 2019. 

Based on clinical evidence and preliminary controlled studies, it has been suggested that cannabis-based medication may be a new and promising treatment strategy for patients with TS. Several clinical studies have been initiated to further investigate the efficacy and tolerability of different cannabis-based medications in the treatment of patients with TS including nabiximols (Sativex^®^) [224], THC (dronabinol) [225,226], and medicinal cannabis [223].

### 5.6. Nausea and Vomiting

Chemotherapy-induced nausea and vomiting (CINV) is one of the most distressing and common adverse events associated with cancer treatment [227]. The CB1 and CB2 receptors have been found in areas of the brainstem associated with emetogenic control [158,228]. Pre-clinical studies suggest that the anti-nausea and anti-emetic properties of cannabinoids (THC, dronabinol, nabilone) are most related to their actions at CB1 receptors [229,230,231]. In addition, an in vitro study suggests that ∆9-THC antagonizes the serotonin receptor (5-HT_3_), a target of standard anti-emetic drugs [232].

Cannabinoids have been used effectively for treating CINV since 1985. In a systematic review, Whiting et al. [103] reported 28 studies (1772 participants) that assessed the use of cannabinoids for nausea and vomiting due to chemotherapy. Fourteen studies assessed nabilone, 3 for dronabinol, 1 for nabiximols, 4 for levonantradol, and 6 for THC. Other specific cannabinoids were not discriminated and individually evaluated. These trials included placebo controlled or used the antiemetics (prochlorperazine, chlorpromazine and domperidone) as comparators. The authors concluded that all trials suggested a greater benefit for cannabinoids than for both comparator and placebo, although the differences did not reach statistical significance in all studies [103].

Dronabinol and nabilone were approved by the FDA in 1985 for the treatment of CINV in patients who have failed to respond adequately to conventional antiemetic treatment [233,234]. The American Society for Clinical Oncology Expert Panel on Antiemetics recently issued updated guidelines and recommended dronabinol and nabilone for the treatment of nausea and vomiting caused by chemotherapy or radiation therapy [235].

Clinical experience remains insufficient for recommendation of cannabis for the treatment of nausea and vomiting caused by chemotherapy or radiation therapy. An uncontrolled pilot study reported that fifty-six patients who had no improvement with standard antiemetic agents were treated with cannabis and 78% demonstrated a positive response to marijuana [236].

Musty et al. [237] reported that patients who used a THC capsule experienced 76–88% relief from nausea and vomiting while those who smoked cannabis showed a 70–100% relief [237]. While a clinical trial comparing ondansetron to smoked cannabis (in doses of 8.4 mg or 16.9 mg THC; 0.30% cannabinol; 0.05% CBD) showed that both doses of Δ^9^-THC reduced subjective ratings of queasiness and objective measures of vomiting; however, the effects were very modest compared to ondansetron [238]. 

The insufficient clinical evidence of medical cannabis combined with the relatively unfavorable side effect profile and the lack of trials comparing cannabinoids with newer antiemetics has limited their clinical utility.

In addition, because medical and legal concerns, the use of medical cannabis and CBD oil is not recommended for management of CINV and is not included in the most recent guidelines for CINV from the Multinational Association of Supportive Care in Cancer (MASCC)/European Society for Medical Oncology (ESMO) and the American Society of Clinical Oncology (ASCO) [235,239].

### 5.7. Treatment of Loss of Appetite 

Weight loss and anorexia can be considered as common secondary side effects of several diseases, such as cancer, and represent a frequent clinical manifestation in patients with human immunodeficiency virus infection (HIV) and advanced acquired immunodeficiency syndrome (AIDS) [102]. This means that when cannabis or its derivatives are implemented, they can improve effects such as pain but can also influence the effect of loss of appetite or weight.

Two systematic reviews were conducted on trials implementing therapies with cannabinoids in patients with HIV and AIDS. Whiting et al. [103] evaluated four randomized controlled trials using dronabinol, with one of them investigating inhaled cannabis as well. These authors concluded that there was some evidence suggesting that cannabinoids were effective in weight gain for patients diagnosed with HIV. Lutge et al. [240] have focused their review on weight and appetite changes in patients with HIV or AIDS in trials in which dronabinol or inhaled cannabis have been implemented. These studies concluded that patient weight increased at higher doses of dronabinol and cannabis and that the median weight also increased with consumption of these compounds when compared to placebo. However, these investigators concluded that evidence on the use of cannabis and cannabinoids in relation to efficacy, safety and changes in appetite and weight were limited and insufficient. 

More recently, Badowski and Yanful [241] performed a review where they compiled information on the use of oral dronabinol in controlling anorexia and weight loss in patients with HIV, AIDS and cancer. This natural compound exerts the effects acting directly on the appetite and vomiting control centers, being indicated in adult patients with HIV and AIDS but not being approved in cancer cases. The authors concluded that there is a limitation in the standardized definitions and that anorexia and weight loss can be misjudged when present in these diseases, and dronabinol can be considered as an additional treatment option in patients. Once again, it was also concluded that with legalization, new studies and new research should be carried out to evaluate the efficacy and safety of cannabis and dronabinol in appetite stimulation.

Andries et al. [242] carried out a study with the aim of investigating the effects of dronabinol treatment on weight and eating disorder-related psychopathological personality traits in cases of women with severe and prolonged anorexia nervosa. The study was conducted at a specialist care center for eating disorders, which included twenty-five women over the age of 18 and diagnosed for at least 5 years, where a randomized study was led between dronabinol-placebo and placebo-dronabinol. The authors concluded that although the sample size was low, there was good tolerance to dronabinol therapy during the four weeks of study, which induced significant weight gain, while no serious psychotropic adverse events were observed. They also concluded that this compound administered at low doses can be used as a safe palliative therapy in diagnosed and selected women.

Finally, Scheffler et al. [243] investigated long-term changes in body mass index, among other parameters, in cannabis users compared to non-users. This study included 109 patients who were treated for various levels of schizophrenia from one episode to patients receiving antipsychotics for 12 months. In contrast to the other reported studies, the researchers that during the first year of treatment there was a greater increase in body mass index in cannabis negative cases compared to cannabis positive cases, after adjusting the different parameters. These differences were not adequately explained by differences in sex, age, alcohol or methamphetamine use, dose or duration of treatment. In contrast to the use of acute cannabis that stimulates appetite, it can be hypothesized that chronic cannabis use may have the effect of suppressing appetite and thus avoiding the weight gain of its users and reducing the risk of obesity. However, other factors such as food malpractice and smoking may influence and contribute to these outcomes. The authors emphasize once again the importance of further longitudinal studies to evaluate possible effects varying in dose and composition of cannabis, with genetics and aspects of quotidian life and in determining the mechanisms by which cannabis reduces the weight gain, being unlikely that it will be used for this purpose.

### 5.8. Cutaneous Treatments and Dermatology

Over the past years, cannabinoids grew interest in the dermatologic area, mostly due to their anti-inflammatory and immunosuppressive properties, as well as its hydrophobic behavior [244]. Some of the research focus on pathologies such as skin cancer and inflammatory skin diseases as contact dermatitis and atopic dermatitis, amongst others [245]. However, according to Theroux and Cropley [246], Piffard was one of the pioneers in this field, leading to further investigation in the present and future, thus suggesting that this theme is not so recent after all. Despite that, the studies covered in this review are recent.

Wilkinson and Williamson [247] conducted a study where the main goal was not only to analyze if phytocannabinoids (THC, CBD and cannabigerol) can inhibit keratinocytes hyper-proliferation (one of the reasons why psoriasis occurs), but also if there is any connection with cannabinoid receptors. In fact, the authors made two important findings. The first is that all cannabinoids tested could indeed inhibit in vitro keratinocytes proliferation. IC_50_ values of maximum inhibition of proliferation for all compounds was reached between 3 and 5 mM, apart from cannabigerol, that was 2.5 and 3 mM. The second is that this mechanism is autonomous from CB1 and CB2 agonist receptors activation. Hence, their hypothesis was that the anti-proliferative effects observed were due to the interaction between cannabinoids and peroxisome proliferator-activated receptor-gamma (PPAR-γ) [247]. The authors observed that cannabigerol and CBD revealed greater potency regarding anti-proliferation, and that the greatest IC_50_ was observed for THC. Still, these results show a potential in using cannabinoids to treat psoriasis.

A different pathology is sebum’s excess and acne. In 2015, Ali et al. [248] conducted a clinical trial where they assessed the effects of a 3% cannabis seed extract cream on 11 males who suffered from acne. By applying such formulation on the subjects’ right cheek twice a day for 12 weeks, culminated in a reduction of sebum level after 48 h, in contrast to the control (cannabis free formulation) on the left cheek. In addition, results show a decrease in skin erythema. Most importantly, no side effects were observed, which sounds promising for future treatments. 

Callaway et al. [249], performed a comparative clinical trial where patients ingested hempseed oil, suggesting this consumption can improve atopic dermatitis. A daily ingestion of 30 mL of the oil showed improvement on skin dryness, irritation, and itchiness, observing a reduction of these symptoms. The group of researchers believes that this results from the high content of polyunsaturated fatty acids present in the hempseed oil. It has been demonstrated that commercial hempseed oil is rich in CBDA, THCA, CBD, THC, CBG, CBN and CBDV [250]. However, Callaway et al., [249] have not studied which of the latter could be responsible for the outcome.

In 2007, a therapeutic clinical trial was carried out in by Eberlein et al. [251] using an endocannabinoid, namely N-palmitoylethanolamine, in a cream used as a regular treatment to assess atopic dermatitis and its symptoms. At the end of the trial, a 58.6% combined score was achieved in what concerns a decline in overall symptoms—erythema, pruritus, and dryness, amongst others. This comes to prove that it is possible to perceive with studies of this kind, in order to reduce corticosteroids as a route of treatment in diseases as atopic dermatitis. 

In a three case-study report, patients autonomously made use of formulations with cannabinoids in its composition, in the form of cream, oil, and spray, in order to treat epidermolysis bullosa, a genetic skin disorder that causes blisters and skin erosion. All three, expressed a decrease of pain and blister’s formation, as well as wound healing [252]. This is beneficial, since it avoids the use of other medicines; however, it is necessary to proceed with further investigation, because this observational study did not take enough further conclusions. 

Another study and a different pathology—Kaposi sarcoma—was studies and it was proved by Maor et al. [253] that CBD can induce cancer cells’ apoptosis, the mechanism is associated to a G protein-coupled receptor that binds to CBD.

Armstrong et al. [254] proved that the induction of autophagy can make THC trigger apoptosis of melanoma cells, leading to loss of carcinogenic cells’ viability. Also, by administrating a “Sativex-like” admixture of equivalent quantity of THC and CBD leads to a less harmful environment, since it prevents melanoma from progressing and inhibits tumor growth. This Sativex^®^ formula is provided as a great example to treat metastatic melanoma, nonetheless further studies should be performed in humans [254]. 

Amongst other herbs, cannabis was included in a study review by Li et al. [255] in which they scrutinized the use of herbs as a medicine for skin cancer. They referred to an in vitro study where anandamide, 2-arachidonoylglycerol, and N-palmitoylethanolamine were used and they observed a reduction of cell viability. 

In 2017, Maida et al. [256] observed cases of patients with pyoderma gangrenosum, a case of inflammatory dermatosis in which patients experience extreme and sometimes chronic painful ulcers. In this study, three patients were observed and accepted to be part of the trial. Patient 1 had applied in his wounds an oil (5 mg/mL of THC + 6 mg/mL of CBD), which was commercially acquired. A daily dose of 1 mL of such oil applied to wounds and bandaging made the patient experience relief of pain and skin irritation, which led to stoppage of the use of corticosteroids. Patient 2, used the same oil with different ratios (7 mg/mL of THC + 9 mg/mL of CBD), applying 0.5–1.0 mL to wounds two/three times a day. As a result, patient experienced a reduction of pain symptoms, nonetheless not statistically significant (*p* = 0.0720). Finally, patient 3 used the same oil composition of patient 2 and applied the same amount (0.5–1.0 mL) two times a day. This patient also experienced pain relief [256]. This research brings out new perspectives for cannabis as an analgesic. However, further studies should be performed, since the sample does not permit to take general conclusions. 

In general, the obtained results bring hope to the potential of cannabinoids as a therapeutic in dermatology. Still, a lot more needs to be done and more clinical trials should be performed. As said before, researches in this field are becoming more frequent. That is why, several reviews on the topic were performed [245,257,258]. 

### 5.9. Infectious Diseases

Pathogenic microorganisms can cause infectious diseases that can spread from one living organism to another, which includes animals and humans. Therefore, there is an urgent need to find solutions to eradicate such diseases. Even though cannabis is being recently investigated as a treatment for many other disorders, when it comes to infections studies they are scarce. In spite of this, there is a case in which CBD was tested as a neuroprotective agent in patients with cerebral malaria [259]. Campos et al. [259], were able to prove in a murine model that CBD can serve as an adjuvant treatment, helping on behavior, relief of anxiety, and improvement of the cognitive function, since the cannabis secondary metabolite promotes recovery of cognitive deficits. In addition, it helped by increasing survival. In any case, these results imply a potential role of cannabinoids as a treatment option, further studies need to be undertaken, to determine cannabinoids’ full potential in this field.

Meza and Lehmann [260] hypothesized that the phytocannabinoid betacaryophyllene (BCP) could activate the cannabinoid type 2 receptor (CB2R) (part of the endocannabinoid system) bettering the beginning of hyper-inflammatory phase on patients with sepsis. The CB2R activation allows an immunosuppressive response, which plays a crucial role in such a phase. As result, it is believed that sepsis aggravation will be reduced. Nevertheless, this study is just a proposal, and further studies need to be pursued to achieve solid and accurate results. 

As observed, studies correlating cannabis as a potential therapeutic target to treat infections are scarce. More needs to be performed in order to achieve significant conclusions. 

### 5.10. Glaucoma

Glaucoma is often caused by an anomalously high intraocular pressure (IOP) on the eye [261]. This pressure can be controlled, therefore for most treatments the main goal is to decrease it [261]. 

In 1998, a review performed by Green [262] came into realization that oral or topical cannabinoids administration would be a good option for glaucoma therapy. However, the same did not happen for smoked marijuana. In spite of IOP reduction on smoking marijuana users, its toxic effects surpassed what was supposed to be beneficial. Nonetheless, only THC is mentioned, and the author defended that further investigation regarding cannabinoids should be done.

In 2006, a trial with six participants was conducted by Tomida et al. [263]. Five milligrams of THC was administrated sublingually and had an effect on the reduction of IOP. Contrarily , 40 mg of CBD increased IOP. Overall, studies are promising, regarding sublingual route administration of THC. However, taking into account that TCH mechanisms on reducing IOP is not yet fully understood, further research and studies need to be performed. 

A recent study by Miller and colleagues [264] tested two phytocannabinoids on mice. THC and CBD were applied atopically. Results showed that 5 mM of THC reduced IOP at a rate of 30% for at least 8 h. The mechanism is associated with cannabinoid-related receptors (CB1 and GPR18). Diversely, CBD was counterproductive, because it diminished THC effects, which was the opposite desire outcome [264]. 

Regardless of the few and redundant studies on this topic, it is possible to conclude that topical and oral administration of cannabinoids have a potential to become part of anti-glaucoma treatment, encouraging research to pursue such an agenda in the field. 

### 5.11. Sleep and Anxiety Disorders

The therapeutic use of cannabis to treat insomnia is a bit controversial, since this is one of the symptoms usually associated to the consumption of this substance. However, in recent years some studies have become very relevant in the therapeutic use of cannabis on people who show sleeping problems. Usually, insomnia is defined as a multifaceted disorder, which is caused by stress factors, health problems that patients may suffer or simply by their daily lives [109]. In some research, it is stated that THC is responsible for sleep promotion, as well as CBD, which have both psychoactive and non-psychoactive properties, which suggests a possible interaction on both cannabis components [265]. Nicholson et al. [266] concluded that THC and CBD have distinct activities on sedative and alerting situations, and therefore their combinations apparently would provide better balance on their activity. Furthermore, some authors claimed that cannabis is effective for insomnia treatment showing results that support the idea of cannabis’ inducing effects on sleep [267]. Nonetheless, it was also evaluated that the use of cannabis would be associated with poor quality of sleep; these contradictory effects found in several studies depend on the concentration of cannabinoids, dose, and the route of administration of this substance [268]. 

Discoveries about the increased consumption of cannabis in people with anxiety problems led some authors to investigate a possible reason for this. The fact that people consume the drug to relax (since relaxation is the most common symptom reported from this drug) may explain this situation [269]. Apparently, THC presents two different effects: when at high doses, the anxiogenic symptoms are predominant, while at low doses the anxiolytic effects prevail. CBD also plays an interesting role in this area, producing anxiolytic effects, and when administered orally has the ability to attenuate the anxiogenic effects of THC. Bergamaschi et al. [270] found that patients who suffer social anxiety disorder had less anxiety when a dose of CBD was provided. This suggests that cannabis use as a medication for anxiety disorders is a very appealing subject for further research. 

To date, studies that were focused on disorders like the Huntington’s or Parkinson’s diseases, dystonia, and also on inflammatory bowel diseases (such as the Crohn’s disease) have not provided scientists with sufficient evidence supporting the effectiveness of cannabis. This situation occurs also with anxiety-related disorders, such as post-traumatic stress disorder; depressive disorders; sleep disorders; and types of chronic pain not yet included in clinical trials as well. Despite that, some patients suffering from these conditions have reported clinical benefits from using cannabis or cannabinoids.

However, for the great majority of these medical conditions, there is either no evidence of effectiveness from controlled clinical trials, or there is limited evidence from studies rated as susceptible to bias because of small patient samples, poor controls or no comparison between cannabis or cannabinoids with placebo or active drug treatments was made [103,261]. Therefore, medical professionals who treat these conditions may be reluctant to use cannabinoids outside clinical trials in the absence of such evidence. Nonetheless, patients are using cannabis and cannabinoids to treat symptoms of these conditions in countries where they are able to do so. This highlights the need to expand the evidence base by undertaking robust studies that cover the full range of cannabis preparations being used, including addressing the issue raised by some patients who report greater benefit from using the whole plant than from using single extracts of cannabinoids, the so-called entourage effect [92,271].

## 6. Challenges in the Determination of Secondary Metabolites of Cannabis

A laboratorial analysis to determine cannabis products use or content can be carried out by measuring of a wide number of cannabinoids and possible metabolites [272]. Nowadays, the most commonly searched cannabinoids on a routine analysis involve cannabigerol, cannabigerolic acid, CBD, cannabidiolic acid, THC, THCA, Δ8-THC, tetrahydrocannabivarin, cannabichromene, and CBN, and their presence can be influenced by several factors. These factors can turn into challenges that can result in erroneous interpretations or incorrect results if not taken into consideration. This analytical determination is of importance, since therapeutic and clinical decisions may depend on it and the assessment of patient compliance as well. In addition, it is also important to evaluate whether the consumption was licit or not, or even to know the concentrations of the active ingredients in formulations. Three big challenges are going to be pointed: (1) sample used for determination; (2) sample preparation; and finally (3) analytical instrumentation, covering the major concerns when cannabinoids are to be determined.

### 6.1. Samples used for Determination

The first big challenge regarding the mentioned cannabinoids is related to the selection of the sample used for their identification and quantification. The analytical field is versatile but requires a careful evaluation according to the main goal of the analysis. The determination of cannabinoids in biological and other specimens has a great importance either in forensic or clinical toxicology, since they are the most widely abused drugs over the world [273]. This analysis can be performed in both biological and non-biological specimens.

#### 6.1.1. Biological Specimens

Blood and urine are historically the elected biological specimens for drug test analysis either in ante-mortem or post-mortem cases [274]. Blood usually identifies a recent drug use and gives an indication of the degree of influence by a drug at the time of collection [272,275]. However, the invasive procedure necessary for its sampling has been pointed as a pitfall. The common alternative is the analysis of urine, applied successfully in workplace drug testing and in abstinence control programs [275,276,277]. Although the presence of certain drugs of abuse or their metabolites in urine can be interpreted as evidence of relatively recent exposure, this does not apply for cannabis [278].

Currently there is an increasing interest for alternative samples to determine cannabinoids, with focus on less invasive collectors and the acquisition of more information regarding cannabis use [274]. These include other body fluids and tissues such as oral fluid, sweat, and hair , or in case of foetal drug exposure, meconium and umbilical cord [272,274]. 

Oral fluid collection is easy and non-invasive, and considered by some authors the only body fluid where drug levels would correlate to those in blood [272,275,278,279,280]. These reasons justified the extensive application of this biological fluid to determine cannabinoids [281,282,283,284,285]. However, the risk of contamination from residual drug in the mouth might not allow this correlation hence this biological specimen has not received broader acceptance to date [272,276]. In addition, its collection may be thwarted by lack of sample available for analysis caused by physiological, or drug itself factors [286]. The presence of food and stimulation techniques might also result in an incorrect quantification of drug presence [286].

The evolution of analytical instrumentation, mainly related to its sensitivity, together with the advances in sweat collection, have made possible the determination of drugs in this specimen [285]. The detection of THC in sweat was reported for the first time in 1990 [287] and later detected in sweat wipes drivers under influence [288,289]. In the past, the scientific community has questioned this matrix’s usefulness since there was a variation between individuals in the amount of sweat they excreted, and the first trials to determine the presence of xenobiotics applied patches that occluded the skin resulting in skin problems (e.g., irritation, alteration of the steady-state, pH, and infections) [274]. More recently, non-occlusive patches are being applied which use an absorbent cellulose pad, being able to detect drug use shortly before the patch was applied and lasting up to seven days [272,275]. Nevertheless, THC is a neutral molecule, unlike the majority of other drugs of abuse, and for this reason, its diffusion is expected to be slower and low amounts are usually detected (ng/patch) [289]. 

The hair drug analysis has become widespread in recent years and thus has become increasingly important [272,290,291,292]. The analysis provides long-term information on drug use over a time period as long as the length of the hair allows (weeks, months, or even years), something that does not occur with other biological matrices [290,292,293]. However, this specimen also presents its pitfalls. One must consider the cosmetic treatments of hair or ethnicity (black or brown hair tend to accumulate more basic-type drugs, due to higher levels of eumelanin) [272]. These factors may lead to an incorrect interpretation of results. In hair, THC was proven to be detected in greater concentrations when compared to THC–COOH, usually the target metabolite to identify cannabis use. This metabolite, although inactive, has a long half-life, but the concentrations found in hair are very low (e.g., sub-picogram per mg range) due to the very poor hair incorporation rate, and sometimes not detectable at all even when extremely sensitive analytical methods are used [294,295]. This may be a problem, since the detection of this metabolite in hair is mandatory to distinguish active consumption from external contamination. In this sense, only sophisticated analytical methods are capable of THC–COOH determination, such as: GC-MS with negative chemical ionisation (NCI) after derivatization with fluorinated reagents; special GC techniques (large injection volume or bi-dimensional-GC); GC-MS/MS; LC-MS, and MS^2^ [290]. In addition, external contamination of THCA has been reported, due to manipulation of cannabis material and side stream smoke [290]. Also, there is evidence of formation of THC resulting from decarboxylation of THCA when alkaline hair digestion at elevated temperatures was applied in sample preparation [290]. Furthermore, the phase I metabolites of THC undergo phase II, forming corresponding glucuronides, and although THC–COOH-glucuronide has been already characterised in hair, the same has not been reported for 11–OH–THC glucuronide [290].

Regarding maternal and neonatal biological specimens, the identification and quantification of drugs and their metabolites offer an objective and reliable approach. Among these specimens, meconium has been widely employed to identify foetal drug exposure during the 3rd trimester [275,296,297]. The main metabolite of THC, THC–COOH, has been reported as the most abundant metabolite detected in meconium samples [298]. The collection of meconium from diapers is also considered non-invasive, which is a big advantage when we are dealing with new-borns, nevertheless some pitfalls have also been pointed, such as the fact that meconium can be expelled prior to, or during, delivery and become unavailable for analysis [299]. In addition, the meconium may not be expelled for days to weeks after birth, and it is often of limited quantity [299]. The determination of cannabinoids in this matrix may be possible without hydrolysing the sample because it has been proven that hydrolysis increases the positivity identification rate [300]. Hydrolysis is known for allowing glucuronides cleavage increasing the free analytes concentration [300]. 

An alternative to meconium regarding foetal drug exposure is the umbilical cord tissue. This specimen is available immediately at birth, for all neonates, and a large amount of tissue is usually available for the analysis [299]. The cannabis data in this sample is scarce and not much information is available on metabolite profile [297].

#### 6.1.2. Non-Biological Specimens

The remarkable pharmacological activity of psychoactive cannabinoids makes drug-type *Cannabis sativa* one of the most researched medicinal plant [301]. However, to the pharmaceutical industry, only two phenotypes are taken in consideration, one being the drug-type cannabis, rich in THC, and used for medicinal or recreational purposes, and other is fibre-type cannabis which is known for being rich in non-psychoactive cannabinoids [302]. The chemical composition of cannabis strains is limited, there is a variability within the plants, interaction of the environment of production, all factors that still require to be carefully monitored [303]. One has to consider that there are a large number of compounds and due to the necessity for standardized treatment in patients (composition and dosage), the use of cannabis should be standardized [304]. Regarding pharmaceutical products, for hemp oil, a great variability was observed for the two samples analysed, although the main component detected was CBD [301]. The authors of this study also point that a decarboxylation process of cannabidiolic acid into CBD might happen for hemp oil and extract during the extraction or during storage over time [301].

In addition, around the world different forms of cannabis products such as, fibres, oils, resins, dried inflorescences, and leaves are consumed [303]. The recent surge in the sale of cannabis-based consumer products has also increased the challenge for sample types required for analysis. These include foods, candies, beverages, topicals, vapes/eliquids, and oral supplements in various forms [305].

### 6.2. Sample Preparation

In any analytical protocol, the sample preparation is crucial in order to achieve accurate and reliable results [273]. The complex nature of the sample matrices, mentioned above, in which cannabinoids may be present, does not allow them to be directly introduced into the analytical instrument [306]. These challenges start with the matrix homogenization and continue through the analyte extraction, clean-up to remove unwanted interferences and derivatization [273].

The traditional liquid–liquid extraction (LLE) and solid-phase extraction (SPE) are commonly the most widely used techniques on a routine cannabinoid determination [272,290,297,298,299,307,308,309,310]. The most common solvent applied in LLE of cannabinoids is mixture of hexane:ethyl acetate on proportions of 9:1 [272,298,307,311] although 1:1 is also mentioned [309]. Other organic solvents might be applied in this technique, such as acetonitrile [310], but the extractions may not reveal as efficient. Regarding SPE, the reported sorbents include mainly mixed mode and cation exchange ones such as Oasis MCX [300], Chromabond HR-XA [290], Strata X-C [281,297], CleanScreen [285], Isolute HCX [312], and specific THC sorbents, such as, CEREX HPSPE THC [299]. These conventional sample preparation techniques result, overall, in great cannabinoids recoveries, nevertheless, several drawbacks have been pointed in the most recent years, namely, difficulty in automation, its complicated and time-consuming processes, and the large amounts of sample and organic solvents that they usually require [313].

The mentioned drawbacks have nowadays been overcome by modern microextraction techniques [273,314,315]. Jain and Singh (2016) [273] have made and excellent and extensive review on these miniaturized procedures applied to cannabinoids determination. Among the most common techniques are solid-phase microextraction (SPME) [316,317,318,319,320,321,322] solid-phase dynamic extraction (SPDE) [323,324], microextraction by packed sorbents (MEPS) [325,326], and dispersive liquid–liquid microextraction (DLLME) [327] among others. These techniques focus on reducing the time of sample preparation and the amounts of organic solvents. Moreover, also trying to reduce the amount of biological specimen required, it reveals advantages such as, low-cost operation, possibility to couple online with analytical instruments and good extraction efficiencies [273,314,315,328].

Regarding the cannabis plants and its commercial products such as pharmaceutical formulations, one should also consider the challenge of maceration and homogenization. As mentioned above, there should be a careful standardization of the method used for cannabis extraction as it is highly important for the introduction of this plant as therapeutic drug [304]. Several extraction solvents have been applied for this purpose. They include ethanol, methanol, chloroform, hexane, petroleum ether, and mixtures of them but in these reports recovery rates are scarce [329]. 

Moran et al. [304] have reported polar solvents as the best suitable to extract cannabinoids, whereas it is most adequate to extract all active compounds with a mixture of polar and non-polar solvents, such as n-hexane and ethanol. These authors focus on FDA regulations that limit the levels of n-hexane in pharmaceutical products, but this can be surpassed by the usage of a more polar solvent such as heptane [304]. On the contrary, Richins [303] uses a less polar solvent, acetone, justifying it as an excellent option for THC with the advantage of extracting fewer sugars and polysaccharides than methanol does. Gul et al. [330] used a mixture of methanol and chloroform (9: 1, v/v) in sample preparation reporting recovery rates above 90% to major components but in the case of cannabigerolic acid, cannabigerol, and Δ8-THC the recovery rates were lower than 85%. 

### 6.3. Analytical Instrumentation

Several analytical methods have been applied to determine cannabinoids in a wide range of matrices. The rising expansion on cannabis market has resulted in a constant development of quantitative analytical methods for their major compounds [305]. Cannabinoids can be detected using immunoassays (EMIT^®^, ELISA, fluorescence polarization, radioimmunoassay), which are usually adopted as a preliminary method in a systematic toxicological analysis (STA). Nevertheless, these can result in false negative and false positive reports which might be consequence of structurally related drugs recognized by the antibodies, adulterants affecting pH, detergents, and other surfactants [331]. In this sense, it is common procedure to confirm by chromatographic techniques any positive result given by the immunoassay [331]. The most common use gas chromatography (GC) coupled to mass spectrometry (GC/MS) [272,285,310,311,312] or flame ionization detector (GC/FID) [303] and liquid chromatography (LC) coupled to mass spectrometry (LC/MS) [301,302,332] or ultraviolet detector (LC/UV) [250,302,305,333]. Nevertheless, GC methods are recognized for its difficulty to identify and quantify acidic cannabinoids such as cannabigerolic acid, cannabidiolic acid, and THCA because they are decarboxylated into their neutral forms during analysis [329,334]. This decarboxylation of cannabinoid acids can be avoided if previously derivatized [335]. Cardenia et al. [335] tested different derivatization procedures with silylation and esterification (diazomethane-mediated), reagents and solvents (pyridine or ethyl acetate), and observed that methylation caused an increase in the signal-to-noise ratio of all carboxylic compounds, except for cannabigerolic acid. In comparison to the GC, the LC appears as a suitable tool to analyze the native composition of the cannabis plant [329]. 

Another challenge related with the analytical instrumentation is their ability to determine these cannabinoids when present in low concentrations. LC coupled to tandem mass spectrometry (LC–MS/MS) using electrospray ionization (ESI) or atmospheric pressure chemical ionization (APCI) is considered nowadays by many authors as the method of choice when drugs of abuse are to be determined, mainly because of the elevated signal-to-noise ratio and selectivity presented [336,337]. For an accurate mass measurement LC coupled with time-of-flight (TOF) MS [299] or hybrid linear ion-trap-Orbitrap (LTQ-Orbitrap) MS [308,338] have been successfully applied to determine cannabinoids [336]. These accurate mass analytical instruments can reveal themselves of crucial importance, together with in vivo assays, for the determination of unknown metabolites.

## 7. Conclusions and Future Perspectives

There is still much to know concerning the effects of most chemical components of cannabis, their efficacy, whether or not patients are responding to the treatment, defining the correct doses, which is the best way of administration, and the side effects. Notwithstanding, the scientific community is becoming more and more interested in studying new therapeutic possibilities of cannabis, despite the fact that there is no scientific evidence supporting its adequate use for most situations. 

Cannabis legalization for medical purposes is still a barrier that needs to be overcome, since in most cases legislation is ambiguous and insufficient to allow regulated access for use in medical situations. Its recreational use concerns authorities, as it is the most consumed drug worldwide; indeed, 200–300 million consumers are estimated around the world. Besides the high number of consumers, in several countries, maximum historical figures were achieved, and there have been several changes in trafficking, with the appearance of high-potency strains of cannabis, edible products, and e-liquids, innovations in the forms of available drug and delivery systems for consumption, which nowadays represent sources of concern for authorities. There is no doubt that further studies are needed to ascertain whether or not the potential benefits compensate the risks, namely in terms of the long-term adverse effects, which are still barely known.
